# Gene-Based Variant Analysis of Whole-Exome Sequencing in Relation to Eosinophil Count

**DOI:** 10.3389/fimmu.2022.862255

**Published:** 2022-07-22

**Authors:** Julia Höglund, Fatemeh Hadizadeh, Weronica E. Ek, Torgny Karlsson, Åsa Johansson

**Affiliations:** Department of Immunology, Genetics and Pathology, Science for Life Laboratory, Uppsala University, Uppsala, Sweden

**Keywords:** exome sequencing, eosinophils, association testing, inflammation, UK Biobank

## Abstract

Eosinophils play important roles in the release of cytokine mediators in response to inflammation. Many associations between common genetic variants and eosinophils have already been reported, using single nucleotide polymorphism (SNP) array data. Here, we have analyzed 200,000 whole-exome sequences (WES) from the UK Biobank cohort and performed gene-based analyses of eosinophil count. We defined five different variant weighting schemes to incorporate information on both deleteriousness and frequency. A total of 220 genes in 55 distinct (>10 Mb apart) genomic regions were found to be associated with eosinophil count, of which seven genes (*ALOX15*, *CSF2RB*, *IL17RA*, *IL33*, *JAK2*, *S1PR4*, and *SH2B3*) are driven by rare variants, independent of common variants identified in genome-wide association studies. Two additional genes, *NPAT* and *RMI1*, have not been associated with eosinophil count before and are considered novel eosinophil loci. These results increase our knowledge about the effect of rare variants on eosinophil count, which can be of great value for further identification of therapeutic targets.

## Introduction

White blood cells (leukocytes) play an essential role in our immune system. Eosinophils (eosinophil granulocytes) are white blood cells that are known to be important mediators of allergic responses. They are phenotypically distinguished from other white blood cells by their bilobed nuclei and large acidophilic cytoplasmic granules. Eosinophils are tissue leukocytes, primarily found in the gastrointestinal tract. Before residing in the tissue, eosinophils are circulated in the bloodstream with a half-life of 8 to 18 h. Therefore, although tissue specimens are required for precise estimation of eosinophil count, enumeration of eosinophils is routinely performed using peripheral blood samples (1). Eosinophils play various complex roles in the body. They are involved in antigen presentation, releasing of cytokine mediators in response to acute and chronic inflammation, reacting to helminth parasites, and homeostasis of the body’s immune responses (1). The number of eosinophils is stringently regulated, and in healthy individuals, eosinophils constitute a small fraction of white blood cells (2). In certain pathologic situations, regulation is perturbed which may lead to a range of clinical consequences. Many allergic reactions, infections, autoimmune disorders, malignancies, and even transplanted organ rejections have been associated with eosinophilia, defined as blood eosinophil count of more than 500 per microliter (1, 3).

Genetic factors have also been postulated to contribute to the variation in eosinophil count between individuals (4). Several hundred genetic associations have previously been reported to eosinophil count (5–8). However, these genetic studies on eosinophil count have primarily investigated the effect of common variants, with the exception of two recent studies investigating rare loss-of-function (LoF) variants in a phenome-wide analysis (9, 10). LoF variants are assumed to be kept at a low frequency due to purifying selection, and previous whole-exome sequencing (WES) studies including only LoF variants have not found any novel genes to be associated with eosinophil count. In the current study, we therefore aim to explore also the effect of rare (not limited to LoF) and common variants combined, in relation to eosinophil count, using WES comprising 200,000 exomes, from the UK Biobank (UKB) (11). Most standard single-marker tests, such as a standard genome-wide association study (GWAS), are generally underpowered in the low allele-frequency domain, especially in combination with potentially low effect sizes (12, 13). For this reason, we extend the analysis to gene-based testing using the sequence kernel association test (SKAT), which is a multivariable regression approach, to better capture the effect of rare variants (14, 15). SKAT can detect associations even if variants have different directions and magnitude of effects, and is less sensitive to including variants with zero effect, in contrast to a burden test. SKAT also allows for the incorporation of weights for individual genetic variants. By assigning weights to variants, their relative importance in the association model is shifted. The two most widely used weighting schemes are by frequency, where rare variants are considered more important, and by deleteriousness, where more harmful variants are considered more important. The CommonRare function in SKAT enables the analysis of common and rare variants as specified by a self-defined minor allele frequency (MAF) cutoff separately. Followed by the combination of the test statistics, this approach has been shown to increase the power when both common and rare variants with effect on the phenotype exist (16). As the underlying genetic architecture is not known and as different genes might have different architectures underlying the variation in the same phenotype, we explored five different weighting schemes in our analysis. We aim to further investigate the role of rare variants altering eosinophil count as this might have considerable implications in the pathogenesis of a wide variety of inflammatory diseases, such as allergic asthma and rheumatoid arthritis.

## Materials and methods

### Study Design

#### Study Cohort and Assessment of Eosinophil Count

The UKB recruited 502,682 individuals, aged 37–73 years, from across the UK during 2006–2010. Most participants were invited once (instance 0), whereas a subset of participants was invited to revisit the assessment center. In the current study, all measurements both for eosinophil count and covariates were extracted from the initial assessment. Eosinophils were measured using hematological assays performed on whole blood using an automated, clinically validated Coulter LH 750 (Brea, CA, USA). Eosinophil count is the proportion of (eosinophils/100) × white blood cell count. Calibration and quality control (QC) were performed in line with the manufacturer’s recommendations. Further details of these measurements can be found in the UKB online showcase and protocol (https://biobank.ndph.ox.ac.uk/showcase/).

Gene-based analyses were performed using the UKB200K WES dataset, in which 200,643 UKB participants have been sequenced. Exomes were captured using the IDT xGEN Exome Research Panel v1.0 (Intergrated DNA Technologies, Iowa, USA) including supplemental probes. Multiplexed samples were sequenced with dual-indexed 75 × 75-bp paired-end reads on the Illumina NovaSeq 6000 platform (Illumina, San Diego, USA) using S4 flow cells. Coverage exceeds 20× at 95.2% of sites on average in each sample and among targeted bases. Reads were then processed and analyzed using the OQFE protocol (https://hub.docker.com/r/dnanexus/oqfe), as described previously (11). Subsequently, initial QC was performed by Regeneron (Thermo Fischer Scientific, Tarrytown, New York, USA) as described in the initial WES data release (9). This included contamination, sex discordance, discordance with microarray data checks, and unresolved duplicate sequences. Following variant QC and extraction of exposures and outcomes, a total of 192,633 participants with WES data and eosinophil measurements were available for analysis.

We performed GWAS on the third release of the UKB genotype data (accessed March 2018). Genotyping has been performed in the UKB using two different custom-designed microarrays: UK BiLEVE and Axiom (Thermo Fischer Scientific, Tarrytown, New York, USA). These contain 807,411 and 820,967 single nucleotide polymorphisms (SNPs), respectively, and overlap with 95% common content. Imputation of over 90 million SNPs was performed by the UKB as well, using UK10K and 1000 genomes phase 3 as reference panels. Before association analysis, we excluded samples with a genetic relatedness with pairwise kinship >0.044, genotype call rate <95%, high heterozygosity, and sex discrepancies between self-reported and genetic sex. Additionally, to avoid possible further population stratification, only participants classified as British Caucasian by both self-identification and by clustering with regard to principal components were selected, leaving 365,954 participants available for analysis.

#### Variant Annotation and Filtering

In all analyses, only autosomal variants were tested. Furthermore, only canonical transcripts (hg38, one isoform per Ensembl gene ID, from here on referred to as gene) as described at the UCSC genome browser were extracted. Variants were then annotated with the Ensemble variant effect predictor (VEP) v99 (17) and Combined Annotation Dependent Depletion (CADD 1.5) (18). A total of 15,886,147 single nucleotide variants (SNVs), 838,879 deletions, and 383,524 insertions were annotated, making it 17,108,550 variants altogether. Variants annotated by VEP as being of high impact (transcript ablation, splice acceptor, splice donor, stop gained, frameshift, stop lost, start lost, transcript amplification; *n* = 435,371) or moderate impact (in-frame insertion, in-frame deletion, missense, protein altering; *n* = 4,403,831) were used for further analysis, resulting in a total of 4,839,202 annotated SNVs and indels used in subsequent analyses.

### Statistical Analyses

#### Gene-Based Analyses

As the main analysis in our study, gene-based combined variance tests on rank-based inverse normally transformed eosinophil count were performed using the SKAT R package (16). Five different weighting schemes were used: 1) SKAT, weighting variants by their CADD value, making predicted deleterious variants become more important in the per variant contribution; 2) unweighted SKAT (β[1, 1]); 3) SKAT, weighting on MAF, making rare variants more important but leaving the contribution of common variants unchanged (Rare: β[0.5, 20], Common: β[0.5, 0.5]); 4) unweighted SKAT CommonRare (Rare: β[1, 1], Common: β[1, 1]); and 5) SKAT CommonRare upweighting rare variants, but in comparison to 3), analyses rare and common variants separate (Rare: β[0.1, 25], Common: β [0.5, 0.5]) (**Figures S13A, B**). SKAT CommonRare first analyzes rare and common variants separately and then combines the test statistics. In the unweighted CommonRare analysis, a default MAF threshold of $\frac{1}{\sqrt{2\times sample\;size}}=\;0.0016$ (0.16%) was used.

In the weighted analysis, we considered a MAF threshold of 0.00025 (0.025%), corresponding to a minor allele count of 100 copies distributed over 400,000 chromosomes (200,000 genomes). In all analyses, age, sex, BMI, smoking status, and the five first genetic principal components (PCs) were used as covariates. We included age, sex, BMI, and smoking status as these have all been shown to influence eosinophil count (19), as well as the five first principal components as they explained 95% of the variability that can be explained by all 40 components. Of the 200,682 participants with WES data available, 8,049 lacked either phenotype or covariate data, resulting in a total of 192,633 participants used in the analyses. The genome-wide significance threshold adjusted for multiple testing was set to $\frac{0.05}{19,288\;\times \;5}$ (i.e., the number of genes times the number of tests), resulting in a Bonferroni-adjusted significance threshold of 5.18 × 10^−7^. As genes located close to each other have a high probability of being genetically correlated, the associated genes located less than 10 Mb from each other were assigned to the same locus. This was done by an iterative procedure, where the gene with the lowest *P*-value for each chromosome was considered to be the lead gene for the first locus of each chromosome, and all significantly associated genes within 10 Mb were assigned to the same locus, iterating until all genes were clustered into a locus. The 10-Mb distance is quite conservative in order not to overestimate the number of independent loci in the gene-based analyses.

#### Single-Marker Analyses

To be able to assess associations that could be captured with a standard GWAS approach in the same cohort, we performed a GWAS on the UKB genotyped data. The association analyses were carried out with plink2 (20), adjusting for the same covariates as the WES data, i.e., age, sex, BMI, smoking status, and the five first principal components. Additionally, only variants with MAF >1% [as imputation quality tends to decrease with decreasing allele frequency (21)], Hardy–Weinberg equilibrium cutoff of 1 × 10^−10^, and a missingness rate of <1% were included. Here, the standard genome-wide significance threshold of 5 × 10^−8^ was used, to be able to capture as many common variants as possible. We then performed conditional analyses, conditioning on the lead variant per locus until no further significant associations were found.

#### Conditional Analyses and Gene-Based Analyses with Different MAF Cutoffs

To examine what type of variant frequency distribution might be driving the driving the gene-based associations, we performed additional analyses. First, we performed conditional analyses on the significant genes from the primary gene-based analyses, adjusting for the lead GWAS hits by adding their dosage values as covariates. To identify if rare variants were likely to drive the SKAT associations, gene-based analyses were performed for the significant genes from the primary analysis, using the RareOnly function in SKAT CommonRare, which only analyzes rare variants below a set threshold. Seven RareOnly analyses were performed setting the MAF cutoff at 0.01%, 0.1%, 0.3%, 0.5%, 1%, 3%, and 5%, respectively, both with and without conditioning on lead GWAS hits. We used the same significance threshold here (*P* < 5.18 × 10^−7^) as in the primary analyses above.

#### Sensitivity Analyses and Meta-Analysis

To improve power, all participants with WES and eosinophil data were included in the primary analysis. As a first sensitivity analysis, we performed additional gene-based tests only including participants that were filtered as in the GWAS (pairwise kinship < 0.044 and classified as British Caucasian by self-identification and by clustering with regard to principal components), both with and without conditioning on lead GWAS hits. This was done to examine whether our results were influenced by possible population stratification.

Second, we performed additional sensitivity analyses. These were performed similarly to the primary analyses in the whole cohort also adjusting for self-reported ethnicity. We performed these analyses both with and without conditioning on GWAS hits. Third, since eosinophil levels are well known to be altered in individuals with allergy or asthma, we performed our analyses similar to the primary analyses but also adjusting for the presence of asthma, hay fever, and eczema. Fourth, to make sure that our choice of five PCs captured enough of the relative variability, we also performed analyses similar to our primary analyses but adjusting for 10 and 15 PCs, respectively.

Lastly, we divided the participants into European (self-identifying as White British) and non-European (self-identifying as Asian or Asian British, Black or Black British, Chinese, Mixed, or Other ethnic groups) and performed the analyses similar to the primary analyses in these two strata. The results were also meta-analyzed using Fisher’s combined probability test. The lowest *P*-value per gene from all weighting schemes per strata was used in the meta-analysis.

#### Intersecting Single-Marker Results and Gene-Based Results

To identify gene-based associations that were overlapping with GWAS results, we compared the gene-based results for overlap with the lead hits from our GWAS. We used bedtools’ (22) “closest” function, which intersects two bed files and reports positional overlap. When no overlap is found, i.e., no lead GWAS SNP was located within the gene region based on start and stop coordinates, it reports the entry (in this case, an SNP) closest to the input entry (in this case, gene position). We defined genes from the SKAT analyses that were located >5 Mb from a lead GWAS SNP as non-GWAS-overlapping. This 5-Mb distance is not to be confused with the 10-Mb distance used to define independent loci from the gene-based results (see above). The independency between all lead GWAS SNPs and the SKAT associations has already been investigated in the conditional analyses (see above).

### Enrichment Analyses and Overlap With Previous GWAS of Eosinophils

To identify overrepresented gene sets, we used Enrichr, a comprehensive gene list enrichment analysis online tool (https://maayanlab.cloud/Enrichr/). We analyzed gene ontology (GO), pathways, and diseases/drugs using the Kyoto Encyclopedia of Genes and Genomes (KEGG) 2021, GO Biological Processes, GO Molecular Function, GO Cellular Processes, ClinVar 2019, GWAS Catalog 2019, and DisGeNET libraries. We used a Bonferroni adjustment of the obtained *q*-values to get a more stringent list of associated pathways, using *q* = 0.05/7 libraries = 0.007 as the threshold for significance.

Lastly, to assess the genes that have been associated with eosinophil count before, they were intersected with all associations to eosinophil count in the GWAS catalog (accessed April 21, 2021). All associations mapping to the trait “eosinophil count” were extracted. In addition to the GWAS catalog, we also verified our results with the database Genebass (genebass.org), described in the preprint by Karczewski et al., where SKAT and burden analyses in 300,000 WES from the UKB are available for more than 3,000 phenotypes (23). The gene-based summary statistics are available online in an interactive browser.

## Results

A total of 192,633 participants with WES data and measured eosinophil count were included in the gene-based analyses. In the GWAS and WES gene-based sensitivity analyses, only unrelated White British participants, *N* = 365,964 and *N* = 143,007 for the GWAS and WES data, respectively, were included. The full workflow is visualized in **Figure S1**. Age at recruitment, the timepoint when eosinophil count was measured, of the participants included in the WES gene-based tests, ranged from 38 to 72 with a median of 58 years, and 55.1% of the participants were women. The mean eosinophil count was 0.18 × 10^9^ cells/L (median: 0.16, range: 0.00–3.24) for men and 0.16 × 10^9^ cells/L (median: 0.12, range: 0.00–5.40) for women. All baseline characteristics can be found in **Table 1**. Of the 17,108,550 annotated variants (**Figure S2A**), a total of 4,839,202 were regarded as “high impact” or “medium impact” as annotated by Ensembl’s variant effect predictor, VEP (**Figure S2B**), and were thus included in the gene-based analyses. To further gain insight into gene-specific variant consequence distribution, we generated gene-specific annotation graphs for genes of interest.

**Table 1:** Baseline characteristics of the UKB participants with exome sequencing data available.

Baseline characteristics			
Number of participants	200,643		
Age, median | 1st–3rd quartiles	58	50–63	
Females | males (%)	110,478 (55)	90,154 (45)	
Eosinophil count, median | 1st–3rd quartiles	0.14	0.10–0.21	
**Inflammatory diseases** ^a^	**Controls**	**Cases**	**% Cases**
Asthma	171,938	28,693	16.7
Hay fever	178,067	22,562	12.7
Eczema	191,674	8,955	4.7
Type 1 diabetes	198,780	1,849	0.9
Psoriasis	198,622	1,996	1.0
Rheumatic arthritis	196,021	4,608	2.4
Crohn’s disease	198,396	2,233	1.1
Ulcerative colitis	199,472	1,157	0.6

a Summary of the inflammatory diseases available in the UKB in which significant genes from our analysis were enriched for the associated genes to the respective disease.

### A Large Number of Associations Captured in a Standard GWAS

We first performed a GWAS for eosinophil count to identify associations, using 6,661,079 common SNPs (MAF > 1%) from the genotyped/imputed data. In the primary analysis, a total of 28,828 significantly associated variants at 106 loci located on all 22 autosomal chromosomes were identified (**Figure S3A**). To ensure that all independent GWAS associations were accounted for in the gene-based analysis, conditional analyses were performed, adjusting for the lead (i.e., the most significant) GWAS hit for each locus until no additional SNPs showed significant association. Across the autosomal chromosomes, a total of 205 lead SNPs, distributed over 106 loci, were identified (**Table S1, Figures S3B–E**). These 205 SNPs were subsequently used as covariates in the downstream conditional gene-based analyses in order to adjust for common variants.

### A Large Overlap Between SKAT Weighting Schemes

**Figure 1:**
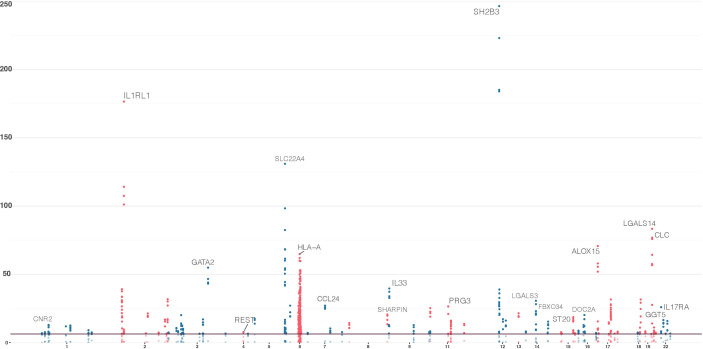
Significant genes in the sequence kernel association test (SKAT) primary analyses; combined results from the five models of the SKAT gene-based analysis. Results from gene-based tests of all canonical transcripts, with chromosomal location at the *x*-axis and −log10(*P*) on the *y*-axis. Genome-wide significance threshold is set at 5.18 × 10^−7^ [−log(*P*) = 6.29]. These 220 genes correspond to the genes that had a significant association with at least one model.

**Figure 2:**
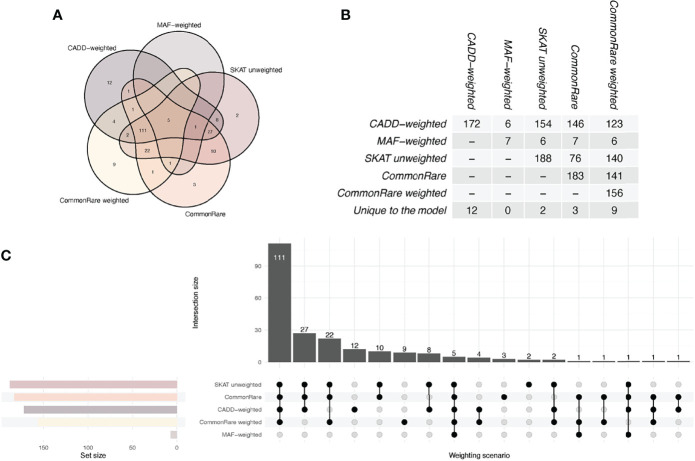
Venn and UpSet diagrams of the overlap between the SKAT weighting schemes. The graph shows the overlap between all comparisons and the table shows the pairwise overlap between the scenarios. CADD, SKAT CADD-weighted; MAF; SKAT MAF-weighted; SKAT, SKAT unweighted; CR, CommonRare unweighted; CR.MAF, CommonRare weighted. **(A)** Venn diagram showing the overlap between weighting schemes, with one color per scheme. The outermost circles show the unique number of hits per weighting scheme, and where the shapes of the schemes overlap, the number of overlapping significant genes is shown. **(B)** Numerical overlap in pairwise comparisons of weighting schemes. The table shows the total overlap, i.e., the summation of each pairwise overlap in **(A)**. **(C)** Overlap visualized as an UpSet plot. The set size represents the total number of significant genes per weighting scheme. The intersection size is showing the number of overlapping genes in each of the respective scheme combinations, as shown by the filled dots underneath.

Across all models, a total of 220 genes, distributed over 19 chromosomes, displayed genome-wide significant association with eosinophil count (*P* < 5.18 × 10^−7^, **Figure 1**, **Table S2, Figures S4–8**). Altogether, the 220 genes represented a total of 55 independent loci (**Table 2**, **Table S3**). We identified only seven associated genes when weighting on MAF. However, in each of the other four models, we identified between 156 and 188 significantly associated genes (**Figure 2**). There was a large overlap in the results between these models with only 12, 2, 3, and 9 associated genes being unique to the CADD, unweighted, unweighted CommonRare, and MAF-weighted CommonRare, respectively (**Figure 2**). Among the 55 lead genes, 3 genes (*IL33*, *ALOX15*, and *S1PR4*) were identified with all weighting schemes, 32 (48%) were identified by four weighting schemes, 10 (18%) by three weighting schemes, and lastly 5 (9%) by both two and one weighting schemes.

**Table 2:** Results for the 55 independent^a^ SKAT loci.

Chr	Lead gene in the locus	Other genes in the locus	Lowest *P*-value^b^	GWAS overlap	Significant gene(s) after GWAS adjustment^c^	GWAS-adjusted *P*-value^d^
1	*TNFRSF8*	*TNFRSF14*, *MIIP*	2.37E−08	No	*TNFRSF14*	3.77E−07
1	*CNR2*	*CEP85*, *FUCA1*, *E2F2*	1.12E−13	Yes	*E2F2*, *CEP85*	4.13E−06, 1.36E−06
1	*ADGRL4*	None	1.29E−12	No	*ADGRL4*	9.00E−08
1	*CCDC18*	*TMED5*	2.73E−13	No	None in locus	0.13
1	*ARNT*	*HORMAD1*, *CTSS*, *ARNT9*	8.06E−10	No	None in locus	0.07
1	*NDUFS2*	None	3.66E−08	No	*NDUFS2*	6.31E−06
2	*IL1RL1*	*ANKRD36C*, *GPAT2*, *ADRA2B*, *ASTL*, *FER1L5*, *IL1RL2*, *IL18RAP*	2.96E−177	Yes	None	0.02
2	*PRKRA*	*ITGA6*	4.78E−22	No	None	0.76
2	*IKZF2*	None	4.55E−08	Yes	None	2.92E−04
2	*D2HGDH*	*GAL3ST2*, *FARP2*, *SCLY*, *INPP5D*	1.77E−32	Yes	None	0.02
3	*IL5RA*	None	4.36E−08	Yes	None in locus	2.38E−03
3	*ACKR2*	*AC006059.2*, *CCDC13*, *GLB1*, *EOMES*, *FYCO1*, *CCR3*, *DALRD3*, *C3orf18*	7.79E−21	Yes	None in locus	2.35E−04
3	*CD200*	*CD200R1*, *SLC9C1*, *SENP7*	1.01E−17	Yes	None in locus	0.016
3	*GATA2*	None	1.30E−55	No	*GATA2*	9.99E−27
3	*HTR3D*	None	2.33E−07	No	None in locus	8.05E−05
4	*REST*	None	3.06E−08	Yes	None	0.08
5	*OTULINL*	None	3.06E−07	No	*OTULINL*	2.15E−06
5	*IL7R*	None	2.60E−18	No	None in locus	0.04
5	*SLC22A4*	*IL9*, *TCF7*, *IL13*, *PDLIM4*, *CSF2*, *IL3M*, *FNIP1*	1.11E−131	No	None in locus	0.04
5	*ADRB2*	None	8.85E−28	No	None in locus	0.26
6	*HLA-A*	See below^e^	1.55E−65	No	None in locus	0.02
7	*ABCB5*	None	7.61E−08	No	None in locus	0.09
7	*CCL24*	None	8.61E−28	No	None in locus	0.06
7	*GATAD1*	None	4.15E−11	No	None in locus	0.07
7	*ZC3HC1*	None	2.17E−08	No	*ZC3HC1*	3.54E−06
8	*TNFRSF10B*	None	4.68E−15	No	None	2.05E−05
8	*SHARPIN*	None	2.79E−21	No	None	0.05
9	*IL33*	*JAK2*	2.40E−40	No	*IL33*, *JAK2*	2.14E−21, 6.21E−08
9	*RMI1*	*KIF27*	1.53E−13	No	*RMI1*	1.65E−07
9	*SEC16A*	*STKLD1*	5.51E−09	No	None in locus	1.37E−03
11	*IFITM2*	None	6.03E−26	Yes	None in locus	0.06
11	*PRG3*	*LTBP3*, *EHBP1L1*, *AP5B1*, *MUS81*, *TSGA10IP*, *SART1*	3.30E−27	No	*PRG3*	1.09E−06
11	*NPAT*	None	2.16E−14	No	*NPAT*	1.62E−07
12	*SH2B3*	*PHETA1*, *CUX2*, *TMEM116*	3.57E−247	No	*SH2B3*	5.15E−12
12	*NAA25*	None	3.83E−25	No	None in locus	0.17
12	*FBRSL1*	*SBNO1*	1.29E−12	No	None in locus	0.04
13	*MRPS31*	None	3.53E−22	No	None	5.46E−03
14	*CEBPE*	None	7.81E−09	No	None	1.02E−05
14	*LGALS3*	*DLGAP5*	2.50E−31	No	None	0.08
14	*FBXO34*	None	7.44E−29	No	None	0.06
14	*ASB2*	*RIN3*	4.46E−16	Yes	None	0.07
15	*NDUFAF1*	*MAP1A*	2.45E−08	No	None in locus	4.55E−05
15	*ST20*	*BCL2A1*, *AKAP13*	2.33E−19	Yes	*BLC2A1*	1.94E−03
16	*SOCS1*	*TNP2*	1.68E−09	No	*SOCS1*	1.41E−07
16	*DOC2A*	*IL4R*, *FBXL19*	6.44E−21	No	None in locus	0.58
16	*ZNF668*	None	7.95E−09	No	*ZNF668*	6.20E−06
16	*NFATC3*	*EXOC3L1*	3.66E−07	No	*EXOC3L1*	3.16E−07
17	*ALOX15*	*PELP1*, *ZNF594*, *CTDNEP1*	1.87E−71	Yes	*ALOX15*, *CTDNEP1*	9.73E−07, 1.16E−06
17	*IKZF3*	*GSDMA*, *ARHGAP27*, *SPPL2C*, *MAPT*, *STH*, *KANSL1*, *LRRC37A*, *LRRC37A2*	1.57E−15	Yes	*GSDMA*	1.97E−06
17	*C17orf58*	*BPTF*	8.79E−09	No	None in locus	9.24E−06
18	*SERPINB11*	*SERPINB13*, *CD226*	4.22E−08	No	*SERPINB11*	7.60E−07
19	*S1PR4*	*ARHGAP45*	2.52E−32	Yes	*S1PR4*, *ARHGAP45*	8.76E−12, 7.50E−07
19	*MAST3*	None	5.43E−09	No	None in locus	0.11
19	*LGALS14*	*ZNF568*, *CLC*, *APOE*, *APOC4-APOC2*, *SIX5*, *CD33*	4.57E−84	No	None in locus	0.24
22	*IL17RA*	*GGT5*, *SFI1*, *CSF2RB*, *GTSE1*	1.07E−26	No	*IL17RA*, *CSF2RB*	3.18E−10, 1.47E−06

a Significant genes from any of the SKAT models were clustered into independent loci based on the genomic distance. This was done by an iterative procedure, where the gene with the lowest P-value for each chromosome was considered to be the lead gene for the first locus of each chromosome, and all significant genes within 10 Mb were considered to belong to the same locus. Then, a second lead gene for each chromosome was identified as the most significant of the genes not belonging to any locus, and this procedure was repeated until no additional genes remained.

b The lowest P-value from the five different gene-based models for the lead gene at each locus.

c None: if no genes at that chromosome passed the significance threshold after adjusting for GWAS hits. None in locus: if no other gene in that particular locus passed the significance threshold.

d P-value for the most significant gene at the locus after adjustment. If no gene in the locus was significant after GWAS adjustment, the adjusted P-value for the lead gene is presented.

e The HLA locus contains the following genes: TRIM31, TRIM40, TRIM15, HLA-E, ABCF1, PPP1R18, VARS2, SFTA2, MUCL3, MUC21, MUC22, C6orf15, PSORS1C1, PSORS1C2, CCHCR1, TCF19, HLA-C, MICA, MCCD1, LTA, PRRC2A, BAG6, C6orf47, GPANK1, LY6G5C, CLIC1, VWA7, VARS1, HSPA1L, SLC44A4, EHMT2, SKIV2L, TNXB, PPT2, PPT2-EGFL8, EGFL8, AGER, NOTCH4, TSBP1, BTNL2, HLA-DRA, HLA-DRB5, HLA-DRB1, HLA-DQA1, HLA-DQB1, HLA-DQA2, HLA-DQB2, PSMB8, HLA-DPA1, HLA-DPB1, TAPBP, ZBTB22, ITPR3, IP6K3, TCP11, H1-1, BTN3A2, BTN3A1, BTN2A1, BTN1A1, ZNF322, PRSS16, POM121L2, H2BC13, OR2B2, ZKSCAN4, NKAPL, ZSCAN26, PGBD1, ZSCAN31, ZKSCAN3, ZSCAN12, OR2J3, OR14J1, OR12D3, OR12D2, MOG, and HLA-G.

### Overlap Between SKAT and GWAS Hits

**Figure 3:**
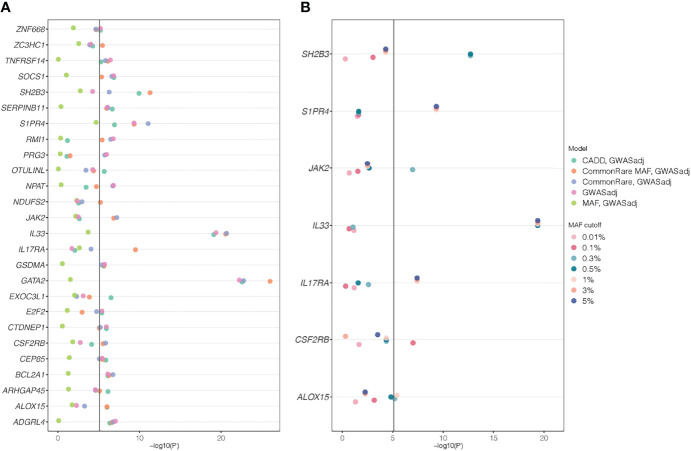
SKAT *P*-values from genes that are significant after adjusting for lead GWAS SNPs. Gene name is stated on the *y*-axes and −log(*P*-value) on the *x*-axes. The different colors depict the different models used in the analyses. **(A)** The five weighting schemes for the 26 genes that were significant after adjusting for lead GWAS SNPs. **(B)** The results when only analyzing rare variants, with different rare variant cutoffs (from 0.01% to 5%) and adjusting for lead GWAS SNPs. Among all 220 genes that were identified in the primary SKAT analyses, only seven genes that were significant for any of the rare variant cutoffs are shown; the others can be found in **Supplementary Table S6**. In all analyses, a *P*-value cutoff of 5.11 × 10^−06^ [−log10(*P*) = 5.29) was used, correcting for the 220 genes times 29 different models tested: seven rare variant cutoffs * two strata (full cohort and unrelated White British) and five weighting schemes for the GWAS-adjusted analyses * two strata and the five weighting schemes for the non-GWAS adjusted (primary/discovery) analyses in the full cohort.

A total of 14 (6.4%) genes at 12 different loci were non-GWAS-overlapping (**Table S3**), based on genomic distance only (>5 Mb away from a lead GWAS SNP). When adjusting for the 205 common lead GWAS SNPs, as many as 26 (12%) out of all 220 genes were still significantly associated in at least one of the models (**Figure 3A**). These 26 genes were located at 21 independent loci, of which six genes (*NPAT*, *RMI1*, *TNFRSF14*, *ADGRL4*, *NDUFS2*, and *ZC3HC1*) at six loci were non-GWAS-overlapping, also based on genomic distance. This suggests that for a majority (62%) of the 55 gene loci identified in the SKAT analyses, the association signals could be explained by a GWAS SNP. The remaining 21 (38%) loci (including the six loci that are non-GWAS-overlapping) remained significant after adjusting for GWAS SNPs, indicating additional association signals, not identified in our GWAS. Among those, six loci included genes (*CEP85*, *GATA2*, *ZNF668*, *EXOC3L1*, *CTDNEP1*, and *SERPINB11*) that had not previously been annotated as eosinophil count genes in the GWAS catalog.

### Sensitivity Analyses

In order to reduce the risk of our results being confounded by population stratification, we performed sensitivity analyses for the gene-based results including only unrelated White British participants. Here, 168 out of the 220 primary genes (**Table S4**), and 21 of the 26 GWAS-adjusted significant genes (**Figure 3B**, **Table S5**), remain significantly associated. Overall, the sensitivity analysis and the main analysis agree well, indicating that including the larger sample size in the unfiltered data can boost the power for discovery. However, due to the reduction in sample size (mixed ancestry, *N* = 192,633; White British only, *N* = 143,077), raw *P*-values are generally higher (i.e., less significant) in the sensitivity analysis due to power reduction (**Figure S9**). We further performed sensitivity analyses, adjusting for ethnicity and occurrence of allergic disease (asthma, eczema, and hay fever), respectively, both with and without conditioning on lead GWAS hits. The results were constant across analyses, indicating that our results are not driven by population stratification or by the inclusion of participants with asthma, eczema, or hay fever (**Table S9**). In addition, the results do not change markedly when increasing the number of PCs included to 10 and 15.

Lastly, we performed two analyses separating participants of European (*N* = 188,248) and non-European ethnicities (*N* = 11,372). We then meta-analyzed the results using Fisher’s method. Adjusting for the 220 primary significant genes, all genes are still significantly associated in the meta-analysis (**Table S10**). Even when adjusting for all lead GWAS hits, the 26 GWAS-adjusted genes remain significant. The non-European sample size is very limited, being almost 17 times smaller than the European, which will influence power. However, *E2F2*, *ZC3HC1*, *RMI1*, *JAK2*, *EXOC3L1*, and *CSF2RB* are nominally significant (*P* < 0.05), also in the small non-European sample, when adjusting for lead GWAS hits (**Table 3**).

**Table 3:** Meta-analysis results for the 26 genes that are still significantly associated in the primary analyses after adjusting for all lead GWAS hits (0.05/220 = 2.3 x 10^-04^).

Chromosome	Gene	*P* Europeans	*P* non-Europeans	*P* meta	*χ*^2^ meta	*d.f.* meta	Adjusted *P* meta^a^
1	*CEP85*	1.98E−06	4.46E−01	1.32E−05	27.88	4	2.90E−03
1	*TNFRSF14*	4.49E−07	6.19E−01	4.47E−06	30.19	4	9.83E−04
1	*E2F2*	3.59E−06	1.39E−03	1.00E−07	38.23	4	2.21E−05
1	*NDUFS2*	7.94E−06	6.19E−02	7.63E−06	29.05	4	1.68E−03
1	*ADGRL4*	1.02E−07	1.47E−01	2.84E−07	36.04	4	6.24E−05
3	*GATA2*	1.31E−26	3.64E−01	2.93E−25	121.22	4	6.45E−23
5	*OTULINL*	1.79E−06	5.01E−01	1.34E−05	27.85	4	2.94E−03
7	*ZC3HC1*	9.47E−06	1.12E−02	1.82E−06	32.11	4	4.00E−04
9	*RMI1*	1.45E−07	9.26E−03	2.89E−08	40.85	4	6.35E−06
9	*IL33*	9.33E−22	2.14E−01	1.02E−20	99.93	4	2.24E−18
9	*JAK2*	6.92E−08	1.85E−02	2.74E−08	40.96	4	6.04E−06
11	*NPAT*	3.65E−07	9.16E−02	6.09E−07	34.43	4	1.34E−04
11	*PRG3*	1.65E−06	1.44E−01	3.85E−06	30.51	4	8.46E−04
12	*SH2B3*	4.28E−12	1.02E−01	1.29E−11	56.91	4	2.84E−09
15	*BCL2A1*	2.52E−09	3.44E−01	1.90E−08	41.73	4	4.18E−06
16	*EXOC3L1*	9.92E−06	1.86E−01	2.62E−05	26.41	4	5.76E−03
16	*ZNF668*	2.63E−07	3.01E−02	1.55E−07	37.31	4	3.42E−05
16	*SOCS1*	1.45E−07	3.57E−01	9.18E−07	33.56	4	2.02E−04
17	*ALOX15*	1.10E−06	9.33E−02	1.75E−06	32.19	4	3.86E−04
17	*CTDNEP1*	1.00E−06	6.74E−01	1.03E−05	28.42	4	2.26E−03
17	*GSDMA*	3.26E−06	1.08E−01	5.57E−06	29.72	4	1.23E−03
18	*SERPINB11*	3.61E−07	1.46E−01	9.36E−07	33.52	4	2.06E−04
19	*S1PR4*	5.18E−12	2.98E−01	4.36E−11	54.39	4	9.59E−09
19	*ARHGAP45*	5.74E−07	1.82E−01	1.79E−06	32.14	4	3.94E−04
22	*IL17RA*	3.09E−10	3.84E−01	2.83E−09	45.71	4	6.23E−07
22	*CSF2RB*	5.28E−06	3.06E−02	2.69E−06	31.28	4	5.92E−04

a Bonferroni-adjusted P-value adjusting for the 220 significant genes tested.*P*-values are shown for Europeans (N = 188,248) and non-Europeans (N = 11,372) as well for the meta-analysis, combining the *P*-values.

### SKAT Associations Driven by Rare Variants

**Figure 4:**
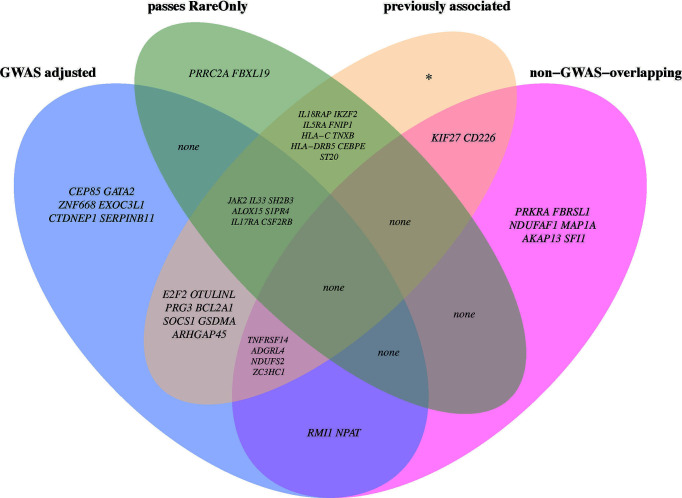
Venn diagram with circles representing four of the downstream analyses. In each section, the overlapping genes are named with their respective gene symbol. If there are no genes in an intersection, “none” is stated. GWAS adjusted: The set of genes that are still significant after adjusting for all lead GWAS hits. Passes RareOnly: The genes that are still significantly associated at any given allele frequency in the analyses including only rare and low-frequency spectrum (<0.01% to <5%). Previously associated: The set of genes that have been previously associated to eosinophil counts in the GWAS catalog. Non-GWAS-overlapping: genes located >5 Mb from a lead GWAS SNP.

To examine whether the associations for primary significant genes were mainly driven by common or rare variants, we performed SKAT CommonRare, only including rare variants (RareOnly). We started with a rare variant threshold of 0.0001 (0.01%) and subsequently increased the threshold to the low-frequency spectrum, stopping at 0.05 (5%). By doing this, 18 genes out of the 220 associated genes showed significant association at least in the low-frequency spectrum (MAF < 1% or MAF < 5%) (**Figure S10, Table S6**). This suggests that these associations are driven by rare variants in combination with common variants. Adjusting for common lead GWAS hits, seven genes (*ALOX15*, *CSF2RB*, *IL17RA*, *IL33*, *JAK2*, *S1PR4*, and *SH2B3*) remained (**Figure 3B**, **Table S6**), all of which were located close to a GWAS hit, except *IL17RA* which is located 3 Mb from the closest lead GWAS SNP. It is therefore plausible that most of these signals represent rare variant associations in addition to the common variant associations. A summary of the MAF thresholds and the number of variants yielding significant associations for these genes can be found in **Table 4**. However, as SKAT does not take gene size into account, one should be cautious when interpreting the raw number of rare variants. None of the non-GWAS-overlapping genes showed significant associations in the RareOnly analysis, implying that these associations are most likely driven mainly or solely by common variants. Taking previously reported associations into account and intersecting our results with these instead, we do find nine genes (*ILRAP*, *IKZF2*, *IL5RA*, *FNIP1*, *HLA-C*, *TNXB*, *HLA-DRB5*, *CEBPE*, and *ST20*) of which seven (*ILRAP*, *IKZF2*, *IL5RA*, *HLA-C*, *TNXB*, *HLA-DRB5*, and *ST20*) show a significant association when only focusing on rare and low-frequency variants (**Figure 4**). This suggests that there might be associations to additional variants that might be of importance in the functional interpretation of the association.

**Table 4:** Summary of the 18 genes that are significant analyzing rare variants only.

Chromosome	Position	Gene	*P*-value	Frequency cutoff	*N* rare variants
2	102,418,689–102,452,565	*IL18RAP*	2.31E−06	0.3%	269
2	212,999,691–213,152,427	*IKZF2*	1.45E−08	0.5%	212
3	3,066,324–3,126,613	*IL5RA*	7.19E−07	0.5%	235
5	131,641,714–131,797,063	*FNIP1*	4.93E−07	0.5%	445
6	31,268,749–31,272,130	*HLA-C*	4.84E−07	1%	479
6	31,620,715–31,637,771	*PRRC2A*	4.14E−07	1%	1,261
6	32,041,153–32,115,334	*TNXB*	6.99E−08	0.5%	2,043
6	32,517,353–32,530,287	*HLA-DRB5*	9.02E−07	1%	77
9	4,984,390–5,129,948	*JAK2*	1.77E−10	0.3%	551
9	6,215,786–6,257,983	*IL33*	1.17E−34	0.5%	153
12	111,405,923–111,451,623	*SH2B3*	3.35E−07	0.1%	429
14	23,117,306–23,119,255	*CEBPE*	7.81E−09	1%	157
15	79,898,840–79,923,702	*ST20*	1.93E−06	0.5%	23
16	30,934,376–30,960,104	*FBXL19*	3.36E−07	1%	255
17	4,630,919–4,642,294	*ALOX15*	4.22E−11	0.1%	364
19	3,172,346–3,180,332	*S1PR4*	5.12E−17	1%	271
22	17,084,954–17,115,694	*IL17RA*	6.89E−08	0.5%	532
22	36,913,628–36,940,439	*CSF2RB*	6.60E−13	0.1%	290

The P-value represents the value corresponding to the lowest frequency cutoff yielding a significant association. Both the allele frequency cutoff and the number of rare variants included in the test are also shown per gene.

### Significant Genes Are Overrepresented in Cytokine-Dependent Pathways

We performed gene set enrichment analyses (GSEA) including the 55 independent lead genes (the most significant gene in each independent loci) as our target, and all the other genes in the genome as the background (**Figure S11, Table S7**). We investigated enrichment with regard to gene ontology, KEGG pathways, and associations to drugs and diseases. For stringency, although the results are FDR-adjusted within each library, we divided the adjusted *q*-value by the number of libraries, ending up in an adjusted *q*-value of 0.007. The most overrepresented ontologies and pathway(s) were all related to cytokine signaling, namely, cytokine–cytokine receptor interaction (KEGG 2021; *Q*_adj_ = 9.04E−04; *IL1RL1*, *IL33*, *CCL24*, *IL15RA*, *TNFRSF8*, *TNFRS10B*, *IL7R*, *IL17RA*), cytokine-mediated signaling pathway (GO Biological Process 2021; *Q*_adj_ = 4.17E−04; *IL1RL1*, *CCL24*, *IL33*, *IFITM2*, *SOCS1*, *ALOX15*, *IL15RA*, *TNFRSF8*, *HLA-A*, *IL7R*, *SH2B3*, *IL17RA*), and cytokine receptor activity (GO Molecular Function 2021; *Q*_adj_ = 2.80E−03; *IL1RL1*, *IL15RA*, *ACKR2*, *IL17R*, *IL17RA*). As expected, the most overrepresented gene sets in relation to traits and diseases were for eosinophil count (GWAS Catalog 2019; *Q*_adj_ = 3.97E−27) and other eosinophil traits, like eosinophil percentage of white blood cells and granulocytes, and white blood cell count. Our lead genes were also enriched for genes that have previously been associated with inflammatory diseases such as rheumatoid arthritis (DisGeNET; *Q*_adj_ = 0.005), psoriasis (DisGeNET; *Q*_adj_ = 0.01), Crohn’s disease (GWAS Catalog 2019; *Q*_adj_ = 0.003), ulcerative colitis (DisGeNET; *Q*_adj_ = 0.02), and allergic disease (GWAS Catalog 2019; *Q*_adj_ = 7.53E−04). Notably, the strongest trait association after eosinophil traits was with asthma (DisGeNET; *Q*_adj_ = 9.13E−05; *CCL24*, *IL33*, *ALOX15*, *IL5RA*, *HLA-A*, *ADRB2*, *GATA2*, *IKZF3*, *IL17RA*, *LGALS3*, *IL1RL1*, *SOCS1*, *NAA25*, *TNFRSF8*, *D2HGDH*, *IL7R*, *CD200*; GWAS Catalog 2019; *Q*_adj_ = 2.13E−04; *IL1RL1*, *IL33*, *D2HGDH*, *IKZF3*).

## Discussion

We have performed an exome-wide scan for genes that are associated with eosinophil count, to identify effects driven by common as well as rare genetic variants. For seven of the identified genes (*ALOX15*, *CSF2RB*, *IL17RA*, *IL33*, *JAK2*, *S1PR4*, and *SH2B3*), we show that rare variants are indeed driving the associations and that these associations were not captured by lead GWAS SNPs. However, these loci are previously known as eosinophil loci, but only for *IL33* rare variant associations have been described in another cohort previously (24). In addition, we identify two completely novel eosinophil loci in our gene-based analyses (*NPAT* and *RMI1*), which are not overlapping with a GWAS locus neither in our present study nor in previous studies, but these do not appear to be driven by rare variants. Together, these novel findings are adding to and extending our knowledge of genetic associations to eosinophil count. However, a large part of our gene-based findings appears to be driven by common variants and overlap with our and previous GWAS results.

Among the genes identified in our gene-based exome analyses, there was a clear enrichment of genes that have previously been associated with inflammatory diseases, including rheumatoid arthritis, psoriasis, Crohn’s disease, ulcerative colitis, asthma, and allergic disease. This is in accordance with elevated levels of eosinophils, eosinophilia, being characteristic of at least allergic inflammation and asthma (25). Our strongest enrichment was seen for asthma with a large number of our eosinophil genes being previously associated with asthma (*CCL24*, *IL33*, *ALOX15*, *IL5RA*, *HLA-A*, *ADRB2*, *GATA2*, *IKZF3*, *IL17RA*, *LGALS3*, *IL1RL1*, *SOCS1*, *NAA25*, *TNFRSF8*, *D2HGDH*, *IL7R*, and *CD200*). We have previously reported that many SNPs that are associated with eosinophil count are also associated with risk for asthma (26), and a genetic correlation has been identified between eosinophils and asthma (27, 28). Even in our sensitivity analyses, when adjusting for the occurrence of asthma, hay fever, and eczema, these genes remain significantly associated with eosinophil count. Altogether, this supports the important role played by eosinophils in the development of inflammatory diseases.

Among our genes that were driven by rare variants, i.e., *ALOX15*, *CSF2RB*, *IL17RA*, *IL33*, *JAK2*, *S1PR4*, and *SH2B3*, previous studies supported a rare variant association between *IL33*, which is a known asthma locus, and eosinophil count (5). When restricting the SKAT analysis to include only rare variants and elaborating with the MAF cutoff for defining a variant as rare, we showed that the *IL33* association is driven by variants with a MAF below 0.5%. This association is most likely mainly driven by rs146597587-C (**Figure S12A**), a splice site-disrupting variant leading to loss of function, that has been shown to be associated with lower blood eosinophil count and to be uncorrelated to previously reported variants in *IL33* associated with eosinophil count (24). Another example is *SH2B3* which seems to be driven by variants below a MAF of 0.3% after adjusting for lead GWAS hits. *SH2B3* has been linked to eosinophil count previously, but to a common missense variant, rs3184504, with a MAF of 48% in the UKB (8). We also verified the association between *SH2B3* and eosinophil count using Genebass. In Genebass, when including missense and low confidence putative LoF variants, an association with *SH2B3* was identified, but not when filtering on high confidence putative LoF variants. The *SH2B3* association is likely partly driven by rs72650673-A (**Figure S12B**, MAF = 0.2%), which has been associated with other blood traits before (8), but not explicitly with eosinophil count.

Our first novel association was *NPAT*, Nuclear protein, coactivator of histone transcription. This gene encodes a protein (NPAT) that is required in the cell cycle through the growth (G1) and DNA synthesis (S) phases, as well as for the S phase entry. In our GSEA, *NPAT* was included in the set of overrepresented genes for both plateletcrit and platelet hematocrit measurement (**Table S7, Figure S11**). Previous studies have also reported genetic associations between rs7129527-G (5), intronic in *NPAT*, and rs4754299-T (8), 2.7 kb upstream of *NPAT*, and plateletcrit. Additionally, in a study of genomic modulators of gene expression in human neutrophils, an SNP (rs35244261) was found to be a cell-type-specific eQTL, being associated with a higher expression of *ATM* in neutrophils and *NPAT* in monocytes (29). As NPAT activates the transcription of histones, there is a possibility that overexpression of *NPAT* in monocytes might give rise to an altered differentiation of myeloid progenitor cells with a higher fraction of monocytes compared to granulocytes (and mast cells and megakaryocytes). Our association between *NPAT* and eosinophil counts might therefore be detected as a result of altered fractions of leukocyte types.

Our second novel eosinophil locus was *RMI1*, RecQ-mediated genome instability 1. The encoded protein (RMI1) is one part of a four-subunit protein complex with BLM, TOPO3, and RMI2. This complex is important during homologous recombination. When this is disrupted, most commonly through LoF mutations in the *BLM* gene, it results in Bloom syndrome. In addition, although *RMI1* has previously not been associated with eosinophils specifically, it has been associated with myeloid white blood cell count. *RMI1* is suggested to be involved in leukemia (including acute myeloid leukemia) where reduced expression of *RMI1* and other critical genes seems to result in disease (30).

Of the genes that overlap with previously reported GWAS results, nine remained significant when analyzing only rare and low-frequency variants. One example is *IL5RA*, coding for the interleukin 5 receptor subunit alpha. One of its ligands, IL-5, promotes the proliferation, differentiation, and activation of eosinophils by binding to the receptor that is located on the surface of the eosinophil (31). IL-5 plays a crucial role in eosinophilic asthma pathophysiology. *IL15RA* has been found to be overexpressed in patients with asthma, albeit only with a moderate correlation to eosinophil count (31). It was also part of sets of overrepresented genes for pneumonia, pneumonitis, eosinophilia, and asthma among others in our GSEA (**Table S7, Figure S11**). IL-5-dependent eosinophil development requires transcription factors from the C/EBP family among others. These are all essential for the commitment and terminal differentiation of myeloid progenitors to the eosinophil lineage (32). *CEBPE*, the gene that encodes the CCAAT enhancer binding protein epsilon, C/EBPϵ, was associated with eosinophil count in our study, also when only considering rare and low-frequency variants. *CEBPE* transcription is driven by two alternative promoters, giving rise to two different protein isoforms with different functions in myeloid differentiation (33). It has been shown that, through the differential usage of these two alternative promoters, the *IL5RA* gene is temporally regulated during eosinophil development. Given that rare variants are often predicted to introduce functional changes, it is plausible that the rare variants driving the associations in our study might be located at critical positions in the gene. However, as our study is gene-based, this warrants further validation.

Six genes, *CEP85*, *GATA2*, *ZNF668*, *EXOC3L1*, *CTDNEP1*, and *SERPINB11*, were still significant after adjusting for lead GWAS hits and have not previously been pinpointed as potential eosinophil genes. One of these, *GATA2*, GATA binding protein 2, encodes a transcription factor that has key roles in hematopoietic development, and its expression has been suggested to identify the early segregation of monocyte and mast cell lineages (34). GATA-2 is known to be involved in early eosinophil differentiation, where overexpression of GATA-2 induces the commitment to the eosinophil lineage in granulocyte–monocyte progenitors (GMPs) (35). As *GATA2* expression seems to be the driver of increasing eosinophil commitment, our results most likely indicate that a change in *GATA2* expression leads to a change in eosinophil count.

As we decided to include participants of all ethnicities, we performed a set of sensitivity analyses controlling for population stratification. Overall, the results agreed well, at least when meta-analyzing the strata representing ethnic groups, and all results are still significant. Adjusting for GWAS hits, all were validated except for *ZNF668*, *ALOX15*, *CTDNEP1*, *GATA2*, and *GSDMA*, which were not genome-wide significant (but still nominally significant) in the analyses when adjusting for lead GWAS SNPs. As our results are reasonably robust across analyses, we believe that these associations, including the novel ones for *RMI1* and *NPAT*, are true association signals, and the changes in the magnitude of the *P*-values are mostly due to changes in sample size.

Our study has some limitations. First, variants used in the GWAS and variants used in the gene-based analyses were not sequenced with the same technology. The third release of genotyped and imputed variants was used in the GWAS, and WES variants were used in the gene-based approach. This makes it harder to accurately interpret the difference in results, especially as imputation is less accurate below 1%. However, as shown by Van Hout et al., *R*^2^ concordance between the 50K WES variants and imputed sequenced data ranged from 32.2% for MAF <0.01% to 95.2% for MAF >1%, with an average of 53.1% across all allele frequencies (9). When compared to array genotypes, concordance was much greater, ranging from 73.2% for MAF <0.01% to 98.7% for MAF >1%, with an average of 92.3% across all allele frequencies. Despite this, one should be cautious with the clinical interpretation, as the variants in the two different datasets were called with different human reference genomes (hg19 for imputed/genotyped and hg38 for WES). It has been shown that there are discordant variants between the reference genomes and that those often are enriched in certain regions (36). Secondly, predicted LoF variants have a high probability of being enriched for annotation errors when residing in the low and rare allele frequency spectrum (37). As the majority of the variants are rare, one should be also cautious with rare variants, especially singletons, that have been predicted as very deleterious. Third, the coverage of the data used in our study (20× at 95.2% of the sites) will have an impact on rare variant detection, even if genotype accuracy has been measured to be as high as 98.5% at 15× coverage, and the improvement of call rate and quality of very rare variants becomes marginal between 15× and 30× depth (38). Lastly, as WES data only contain exonic variation, we might miss associations outside of coding regions, such as regulatory elements or functional variation within introns. Most notably, our results warrant functional validation, especially the previously established associations where we identified additional rare variants that seem to be driving the association, even after adjusting for lead GWAS hits.

A recent study has analyzed exome sequencing data from 450,000 UK Biobank exomes in relation to eosinophil traits (10). However, the previous study limited its analyses to gene burden tests where rare variants were collapsed within a gene region. The collapsing was performed so that individuals who did not have any likely deleterious variant in the region were classified as being homozygous reference, whereas heterozygous carriers of any likely deleterious variant were considered heterozygous, and homozygotes for any likely deleterious variant were considered homozygotes. The same collapsing strategy was done for LoF variants as for the likely deleterious. In our study, we instead used the SKAT method that does not collapse variants in a similar way. In contrast, SKAT does not assume that the effects of the variants are in the same direction and have the same magnitudes of effects. In addition, SKAT also allows for the incorporation of weights for the genetic variants, weights that can reflect the predicted degree of deleteriousness or the MAF. In our study, we have therefore been able to report additional associations that were not identified in the previous study (10). We designed our study to test a set of different weighting schemes in the gene-based analyses and identified a total of 220 genes, of which 5 (2.3%) were identified by all weighting schemes, 112 (50.9%) by four, 53 (24.1%) by three, 24 (10.9%) by two, and 26 (11.8%) by one weighting scheme, respectively. Different weights may be optimal for different regions, but the most optimal analysis method cannot be chosen, as we do not have sufficient information regarding the underlying genetic architecture. Most importantly, as the underlying architecture is most likely not uniform across all causal regions, there will likely never be a “one-size-fits-all” model.

In summary, we have identified two novel loci for eosinophil count, *RMI1* and *NPAT*, and we also report several novel rare variant associations in previously associated genes. Despite this, a relatively low number of associations appears to be driven by rare variants. However, rare variants are more likely to have larger phenotypic effects, compared to common variants, and by that, rare variants are of higher clinical impact. However, even with 200,000 exomes, the number of rare variant associations was limited, which could suggest that even larger sample sizes are needed in order to reach enough statistical power to investigate the effect of very rare variants. Also, one should consider that our study only investigated exonic variants, which is a vast minority of the total amount of variations in the human genome, and there is therefore a future need to expand rare variant analyses to include also a non-coding variation to capture additional associations for eosinophil count.

## Data Availability Statement

The data analyzed in this study is subject to the following licenses/restrictions: The data on which this study is based (application number 15479) are available for bona fide researchers from the UKB Resource, on filing an application to the UKB. Relevant additional data will be available from the authors on request. For further information, please contact the corresponding authors. Requests to access these datasets should be directed to UK Biobank: http://www.ukbiobank.ac.uk/about-biobank-uk; JH, julia.hoglund@igp.uu.se; ÅJ, asa.johansson@igp.uu.se.

## Ethics Statement

The UKB resource was reviewed and approved by the North West Multicentre Research Ethics Committee (covering the United Kingdom), National Information Governance Board for Health and Social Care (covering England and Wales), and Community Health Index Advisory Group (covering Scotland). The UKB possesses a generic Research Tissue Bank approval granted by the National Research Ethics Service. This approval allows applicants to conduct research on UKB data without having to obtain separate ethical approvals. Additionally, the UKB study was reviewed and approved by the National Research Ethics Committee (REC reference 11/NW/0382). The participants provided their written informed consent to participate in this study. An application for using data from the UKB has been approved (application nr: 15479). The UKB analysis performed in this study has also been approved by the Swedish Ethical Review Authority (dnr: 2020-04415).

## Author Contributions

JH, WE, and ÅJ designed the study. JH performed the data analysis and generated the figures. JH and TK performed the statistical analysis. FH performed the literature search and result interpretation. JH and FH wrote the manuscript. JH, WE, FH, TK, and ÅJ interpreted the data and contributed to and reviewed the manuscript. All authors contributed to the article and approved the submitted version.

## Funding

The research was funded by the Swedish Research Council (2019–01497); the Swedish Heart and Lung Foundation (20200687); and the Åke Wiberg, Marcus Borgström, A and M Rudbergs, and Hedströms K and O F Foundations.

## Conflict of Interest

The authors declare that the research was conducted in the absence of any commercial or financial relationships that could be construed as a potential conflict of interest.

## Publisher’s Note

All claims expressed in this article are solely those of the authors and do not necessarily represent those of their affiliated organizations, or those of the publisher, the editors and the reviewers. Any product that may be evaluated in this article, or claim that may be made by its manufacturer, is not guaranteed or endorsed by the publisher.
