# Supplementary material for: Gene-Based Variant Analysis of Whole-Exome Sequencing in Relation to Eosinophil Count
Source: Front Immunol. 2022 Jul 22;13:862255. doi: 10.3389/fimmu.2022.862255 (PMC9355086; doi:10.3389/fimmu.2022.862255)
Supplement: Supplementary file 1 [file DataSheet_1.pdf]

Supplementary Material

1 Supplementary Tables

1.1 Table S1

Results from the GWAS analysis. Lead hits from both primary and conditional analysis are shown. An adjusted P value of 5x10^-8 have been used to capture as many common variants as possible. **Chromosome**: The chromosome on which the variant is located. **Position**: Position in base pairs (GrCh37). **SNP**: The name of the variant. If no rs-id is available, they are called chr:pos_A1_A2. **Other allele**: The other allele (in contrast to the effect allele. always major). **Effect allele**: The effective allele used in the analysis (always minor). **EAF**: Effect allele frequency. **Beta**: Effect. beta estimate. **T statistics**: T statistics for the estimate. **P value**: the variant's P value. **GWAS run**: the analysis number where 1 corresponds to primary analysis. and 2,3,4 corresponds to first, second and third conditional analysis

1.2 Table S2

Results for the 220 genes that passed genome-wide significance in the SKAT gene-based tests for at least one of the five models tested. **Chr.**: the chromosome on which the variant is located. **Start**: start position of the gene (GrCh38). **End**: end position of the gene (GrCh38). **Transcript**: the name of the transcript analyzed. **Gene**: the gene symbol of the associated gene. **N Marker All**: total number of genetic markers within the gene region. **N Marker Test**: total number of genetic markers tested in the model. **N Marker Common**: total number of common variants tested in the CommonRare models. The number outside brackets is the number of common variants in the unweighted CommonRare analysis with a MAF threshold of 0.16% and the one within brackets the number of common in the weighted CommonRare analysis with a MAF threshold of 0.025%. **N Marker Rare**: total number of rare variants tested in the CommonRare models. **SKAT**: the P value for the unweighted SKAT model. A dash means that this model did not yield a significant result for the particular gene. **SKAT CADD weighted**: the P value for the CADD-weighted SKAT model. A dash means that this model did not yield a significant result for the particular gene. **SKAT MAF weighted**: the P value for the MAF-weighted SKAT model. A dash means that this model did not yield a significant result for the particular gene. **SKAT CommonRare**: the P value for the unweighted SKAT CommonRare model. A dash means that this model did not yield a significant result for the particular gene. **SKAT CommonRare MAF weighted**: the P value for the weighted SKAT CommonRare model. A dash means that this model did not yield a significant result for the particular gene.

** The number outside brackets is the number of rare variants in the unweighted CommonRare analysis with a MAF threshold of 0.16% and the one within brackets the number of rare in the weighted CommonRare analysis with a MAF threshold of 0.025%.

1.3 Table S3

Location and information for the 220 genes that passes genome-wide significance with at least one significance model. Independent genes (i.e., independent association signals) as defined by genomic

distance are shown in bold. The gene with the lowest p value has been defined as the primary lead hit. Genomic distance (in base pairs) has then been calculated to the genes nearby. If a gene is located less than 10 Mb from the lead gene, it is considered being located in the same locus. When the distance exceeds 10 Mb, the next gene with the lowest p value is considered as the secondary lead hit. Distances have been calculated from the lead hits until no more independent loci are detected. These have then been considered independent loci. Further, intersection of the lead GWAS hits and the significant gene-based results. All genomic positions are stated in GrCh38. Results from the GWAS were lifted to GrCh38 before intersecting. If the closest lead GWAS hit is located > 5Mb from the closest significant gene, that gene is considered non-GWAS-overlapping. **Chr**: the chromosome on which the variant is located. **Position**: start and end position of the gene (GrCh38). **Transcript**: name of the associated transcripts. **Gene**: the gene symbol of the associated gene. **Lowest P-value**: P value for the most significant of the five SKAT-models. **Passes GWAS adj**: whether or not the gene is still significant after adjusting for all GWAS hits. **Non-overlapping**: whether or not the gene is considered non-GWAS-overlapping. **SNP Position**: genomic position of the GWAS hit (GrCh38, lifted over). **SNP**: rs-id for the GWAS hit. If no rs-id is available they are called chr:pos_A1_A2. **logP**: -log(P) for the GWAS hit. **Lead SNP located within gene**: whether or not the lead GWAS hit was located within the gene. including non-exonic regions. **Distance to closest**: the distance in bp from the associated gene to the closest GWAS hit. A dash means an intragenic hit

* The most significant gene in the locus is stated in bold

1.4 Table S4

Comparison between the results for the 220 significant genes from the main analysis and the sensitivity analysis. **Chr**: the chromosome on which the variant is located. **Position**: start and end position of the gene (GrCh38). **Transcript**: the name of the transcript analyzed. **Gene**: the gene symbol of the associated gene. **Info**: main - primary analysis. sens - sensitivity analysis. **SKAT**: the P value for the unweighted SKAT model. **SKAT CADD weighted**: the P value for the CADD-weighted SKAT model. **SKAT MAF weighted**: the P value for the MAF-weighted SKAT model. **SKAT CommonRare**: the P value for the unweighted SKAT CommonRare model. **SKAT CommonRare MAF weighted**: the P value for the weighted SKAT CommonRare model.

1.5 Table S5

Comparison of the results for the 26 genes still significant after adjusting for all lead GWAS hits, between the primary and sensitivity analysis. Genes in bold passes both primary and sensitivity analysis using an adjusted significance threshold of 5.11E-06. **Chr**: the chromosome on which the variant is located. **Position**: start and end position of the gene (GrCh38). **Transcript**: the name of the transcript analyzed. **Gene**: the gene symbol of the associated gene. **Info**: adj - primary analysis adjusted for all lead GWAS hits; adj. sens - sensitivity analysis adjusted for all lead GWAS hits. **SKAT**: the P value for the unweighted SKAT model. **SKAT CADD weighted**: the P value for the CADD-weighted SKAT model. **SKAT MAF weighted**: the P value for the MAF-weighted SKAT model. **SKAT CommonRare**: the P value for the unweighted SKAT CommonRare model. **SKAT CommonRare MAF weighted**: the P value for the weighted SKAT CommonRare model

1.6 Table S6

Results from the CommonRare rare only analyses at different MAF thresholds for defining rare variants. The 18 genes that had a significant association at least in the low-frequency spectrum of MAF 1-5% are presented. Pre analysis, all gene-sets have already been filtered based on deleteriousness, keeping variants of high and moderate impact. **Chr**: the chromosome on which the variant is located. **Position**: start and end position of the gene. **Transcript**: the name of the transcript analyzed. **Gene**: the gene symbol of the associated gene. **Info**: P value - the P value for the analysis; N Marker Rare - number of rare genetic markers used in the analysis; P value (adj) - P value for the GWAS adjusted analysis; N Marker Rare (adj.) - number of rare genetic markers used in the GWAS adjusted analysis. **RareOnly 0.01%:** results for the RareOnly analysis at MAF threshold at 0.01%. **RareOnly 0.1%**: results for the RareOnly analysis at MAF threshold at 0.1%. **RareOnly 0.3%:** results for the RareOnly analysis at MAF threshold at 0.3%. **RareOnly 0.5%:** results for the RareOnly analysis at MAF threshold at 0.5%. **RareOnly 1%:** results for the RareOnly analysis at MAF threshold at 1%. **RareOnly 3%:** results for the RareOnly analysis at MAF threshold at 3%. **RareOnly 5%:** results for the RareOnly analysis at MAF threshold at 5%

1.7 Table S7

Significant results (FDR < 0.05) from the enrichment analyses with Enrichr GSEA. **Term**: name of the term (pathway, disease, drug, trait. GO term and alike)**. Overlap**: the overlap between the genes in the search query and the total number of genes in the gene set**. P-value**: P value calculated within each gene by the Fishers exact test assuming independent genes**. Q-value (FDR)**: adjusted p value using Benjamini-Hochberg FDR**. Bonferroni Adjusted Q-value**: q values adjusted for multiple testing. i.e., for the 7 different libraries used**. Odds Ratio**: odds ratio of the gene set**. Combined Score**: combined ranking score. i.e., z value multiplied by -log(P value)**. Genes**: the genes included in the set. **Library**: name of the library**. Type**: type of library

1.8 Table S8

Result overlap with previously reported associations to eosinophil counts in the GWAS catalog. If a previous GWAS association is intergenic and have mapped between two genes, one of which was significantly associated in the SKAT gene-based analysis, that gene has been counted as previously associated. If no previously reported association was found, it is stated with an NA. **MAPPED_GENE:** the gene reported in the GWAS catalog. which was used to intersect with the SKAT gene-based results. **DISEASE.TRAIT**: the mapped disease trait. i.e., Eosinophil count. **SNPS**: the reported significantly associated SNPs. **CONTEXT**: the variant annotations for the reported SNPs

1.9 Table S9

Results for the sensitivity analyses adjusting for 10 PCs, 15 PCs, ethnic background and presence of allergic disease respectively. The top part shows the 26 genes that passes the conditional analyses adjusting for all lead GWAS hits. The bottom part shows the sensitivity analyses for all 220 genes that passed the significance threshold in the primary analyses. **Chromosome**: Number of the chromosome. **Transcript**: the name of the transcript analyzed. **Gene**: the gene symbol of the associated gene. **Adj. For 10 PCs**: Analysis adjusted for the first 10 genetic principal components. **Adj. for 15 PCs**: Analysis adjusted for the first 15 genetic principal components. **Adj. for self-reported ethnic group**: Analysis when adjusting for self-reported ethnic group (White, Mixed, Asian or Asian British, Black or Black British, Chinese or Other). **Adj. for allergic disease:** Analysis when adjusting for presence of allergic disease (Asthma, Eczema and/or Hay Fever)

1.10 Table S10

Results for the meta-analysis in Europeans and non-Europeans for all 220 primary significantly associated genes. Results are not adjusted for lead GWAS hits. **Chromosome**: The chromosome on which the gene is located. **Gene**: The gene symbol of the associated gene. **P Europeans**: P value from the analysis in European participants only (N = 188 248). **P non-Europeans**: P value from the analysis in non-Europeans (N = 11 372). **P Meta**: P value from the meta-analysis using Fisher's method. **χ^2 Meta**: Chi-square from the meta-analysis. **d.f. Meta**: number of degrees of freedom per test in the meta-analysis. **adjusted P Meta**: P value adjusted for the 220 significant genes from the primary analysis

2 Supplementary Figures

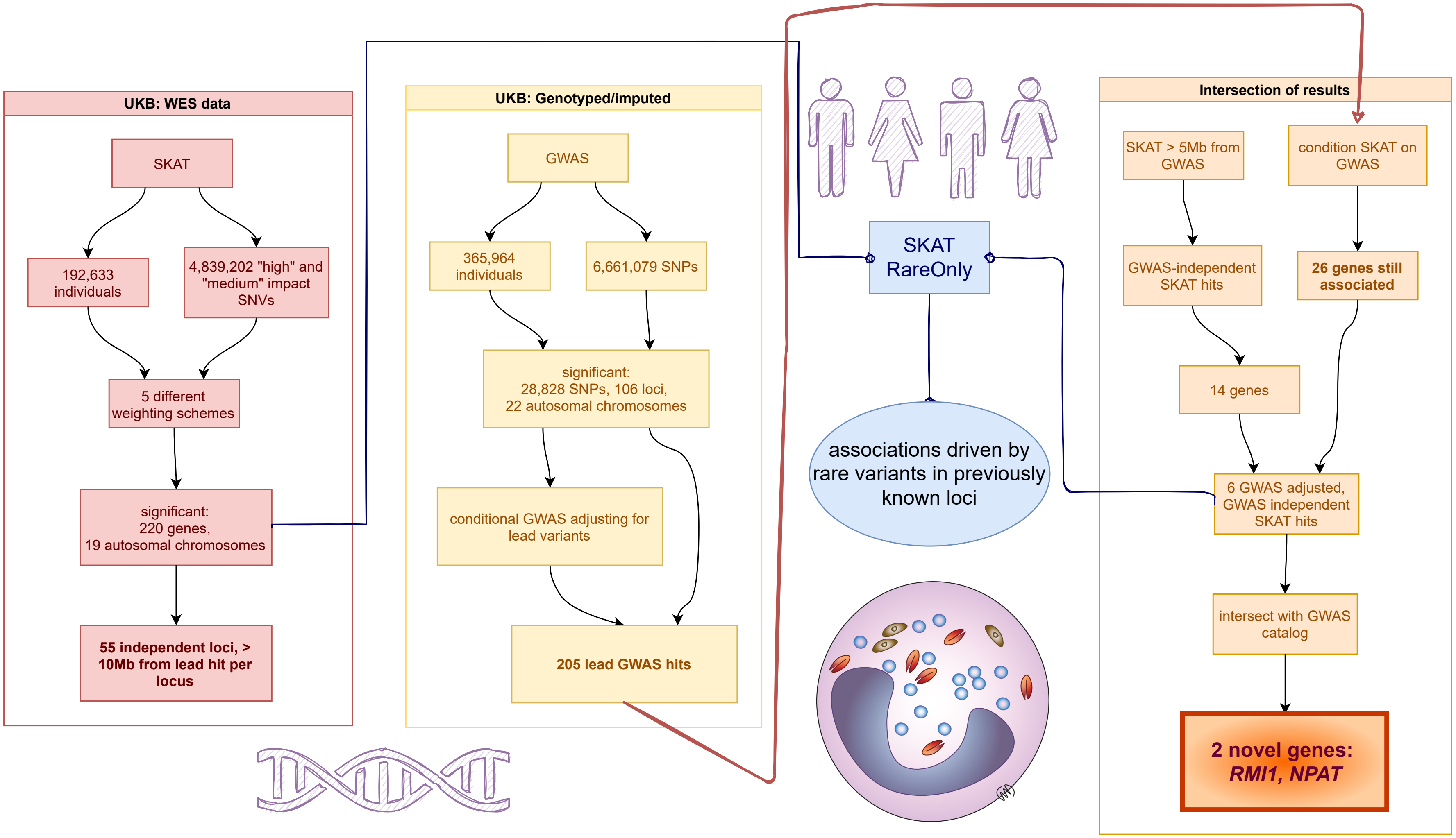

Fig. S1. Study workflow.

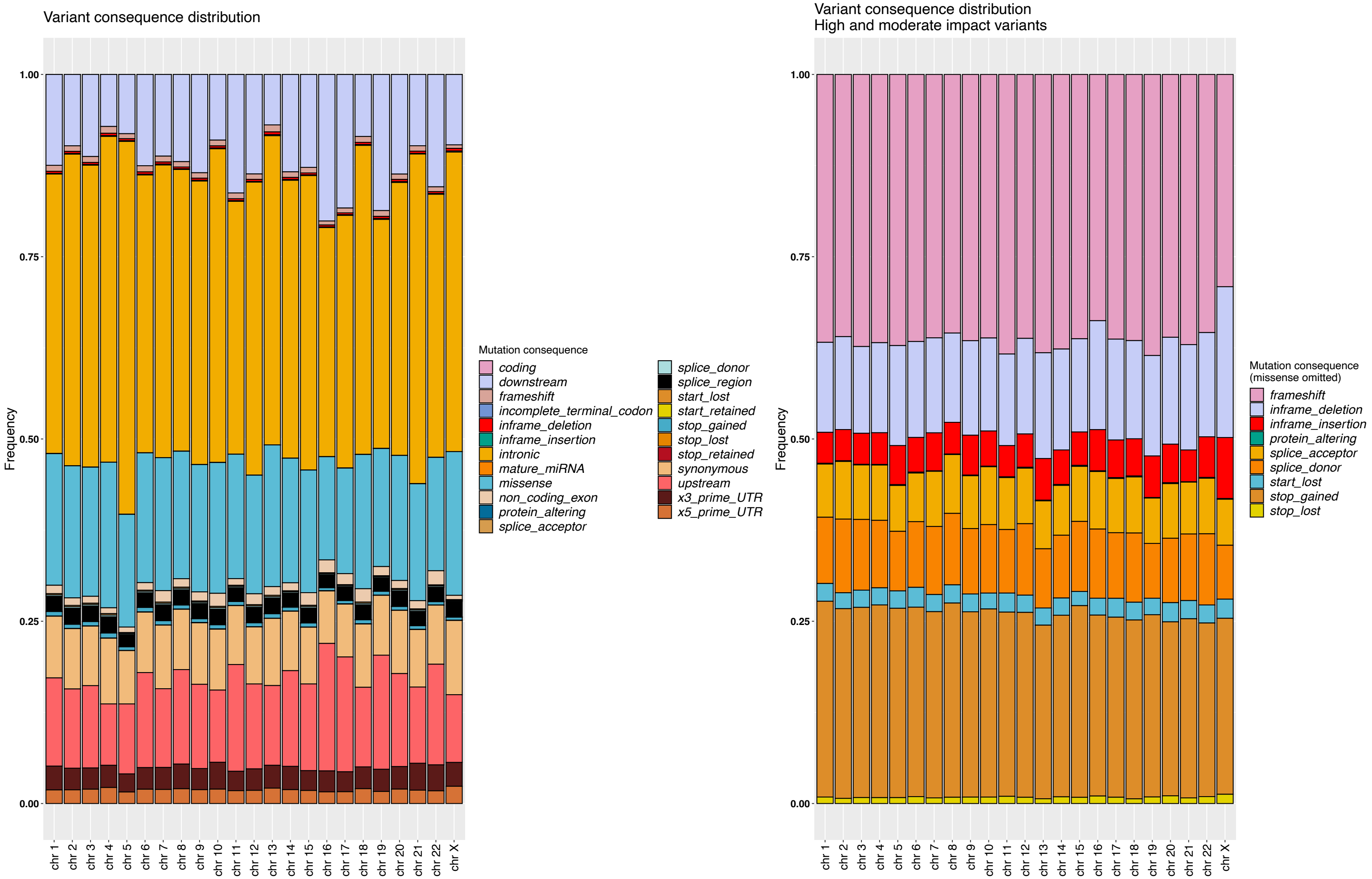

Fig. S2. Variant consequence distribution of the annotated variants in the UKB200K dataset. **a)** Total variant consequence distribution. **b)** Variant consequence distribution among the variants classified as either of high or moderate impact by Ensembl's variant effect predictor, VEP. Missense variants are omitted for better visualization of the less common consequences

frontiers

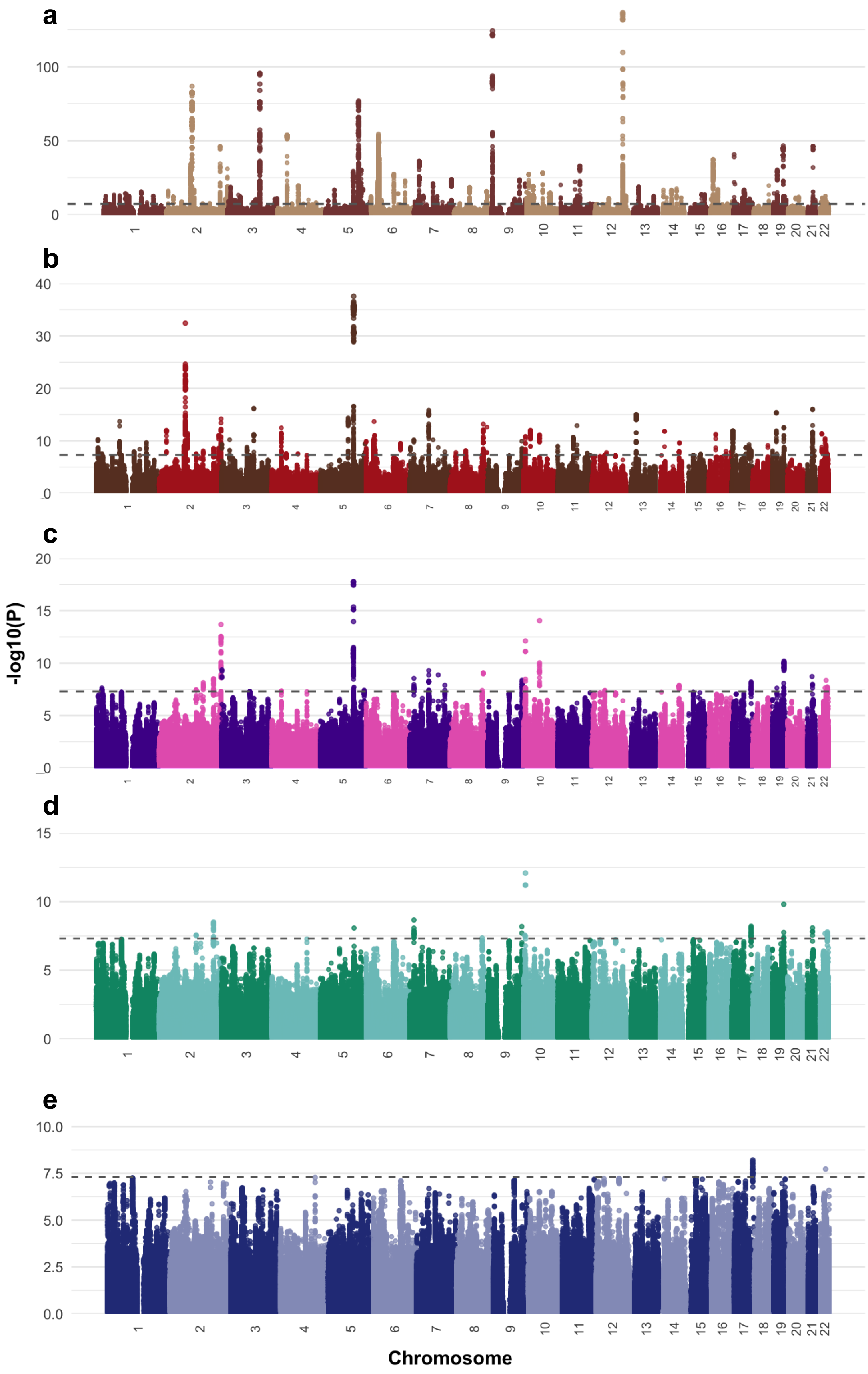

Fig. S3. Manhattan plots depicting GWAS results on genotyped/imputed data. Chromosome number on the x-axis and -log10(P) on the y-axis. The more lenient genome-wide significance threshold of P = $5x10^{-8}$ (-log10(P) = 7.3) was used. **a)** GWAS results from the primary analysis, only adjusting for age, sex, BMI, smoking status and the five first genetic principal components. **b)** GWAS results from the secondary analysis adjusting for covariates and the top GWAS hit at each locus. **c)** Adjusting for covariates together with the top GWAS hit from both the primary and secondary analysis. **d)** Adjusting for covariates together with the top GWAS hits from the three prior analyses **e)** Lastly, GWAS results adjusting for all top GWAS hits from the four prior analyses.

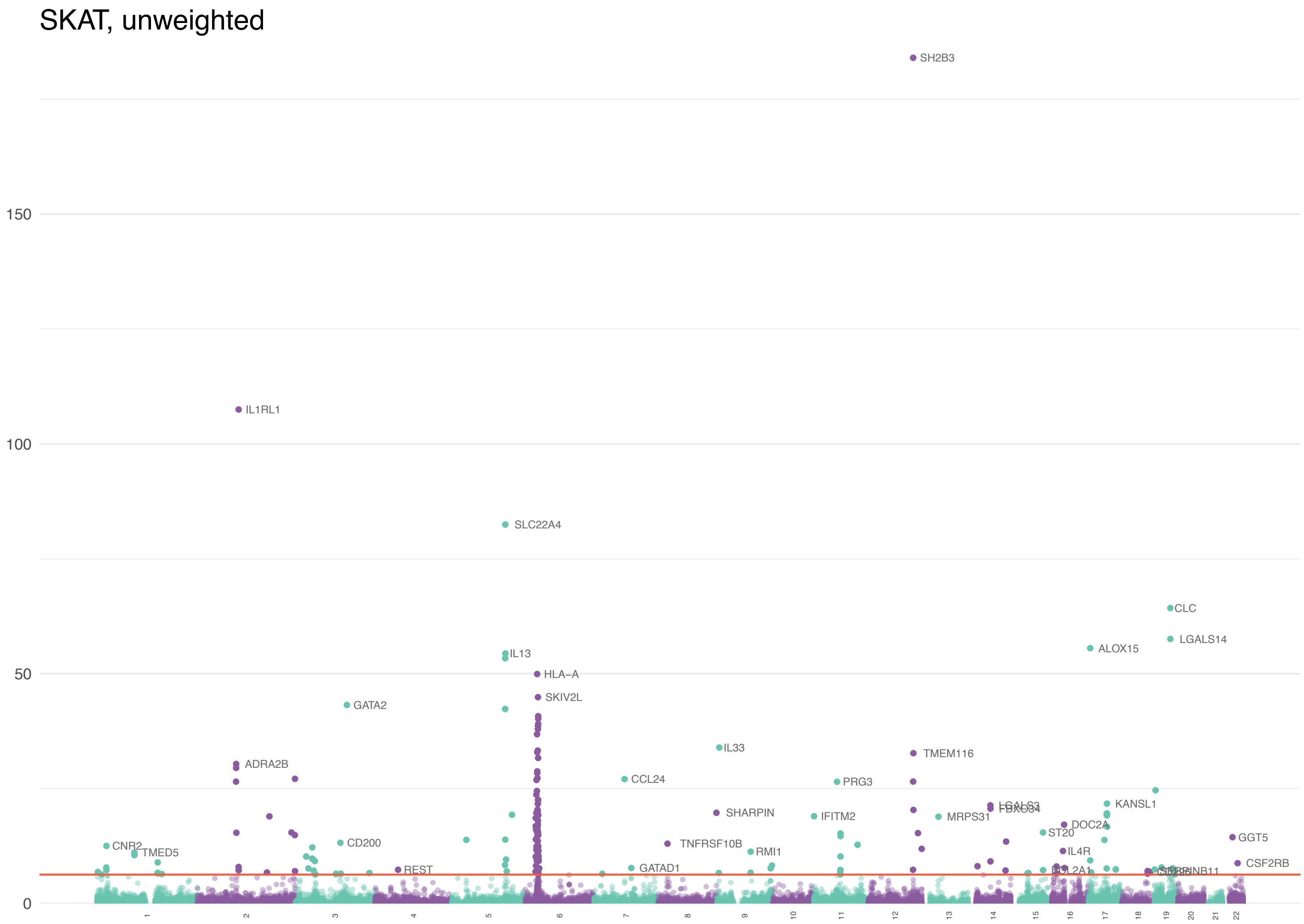

Fig. S4. Results from the unweighted SKAT gene-based analysis. Results from gene-based tests of all canonical transcripts, with chromosomal location at the x-axis and -log10(P) on the y-axis. Genome-wide significance threshold is set at P = 5.18x10^{-7} (-log10(P) = 6.29). The top two most significant genes at each chromosome are labelled with their gene name.

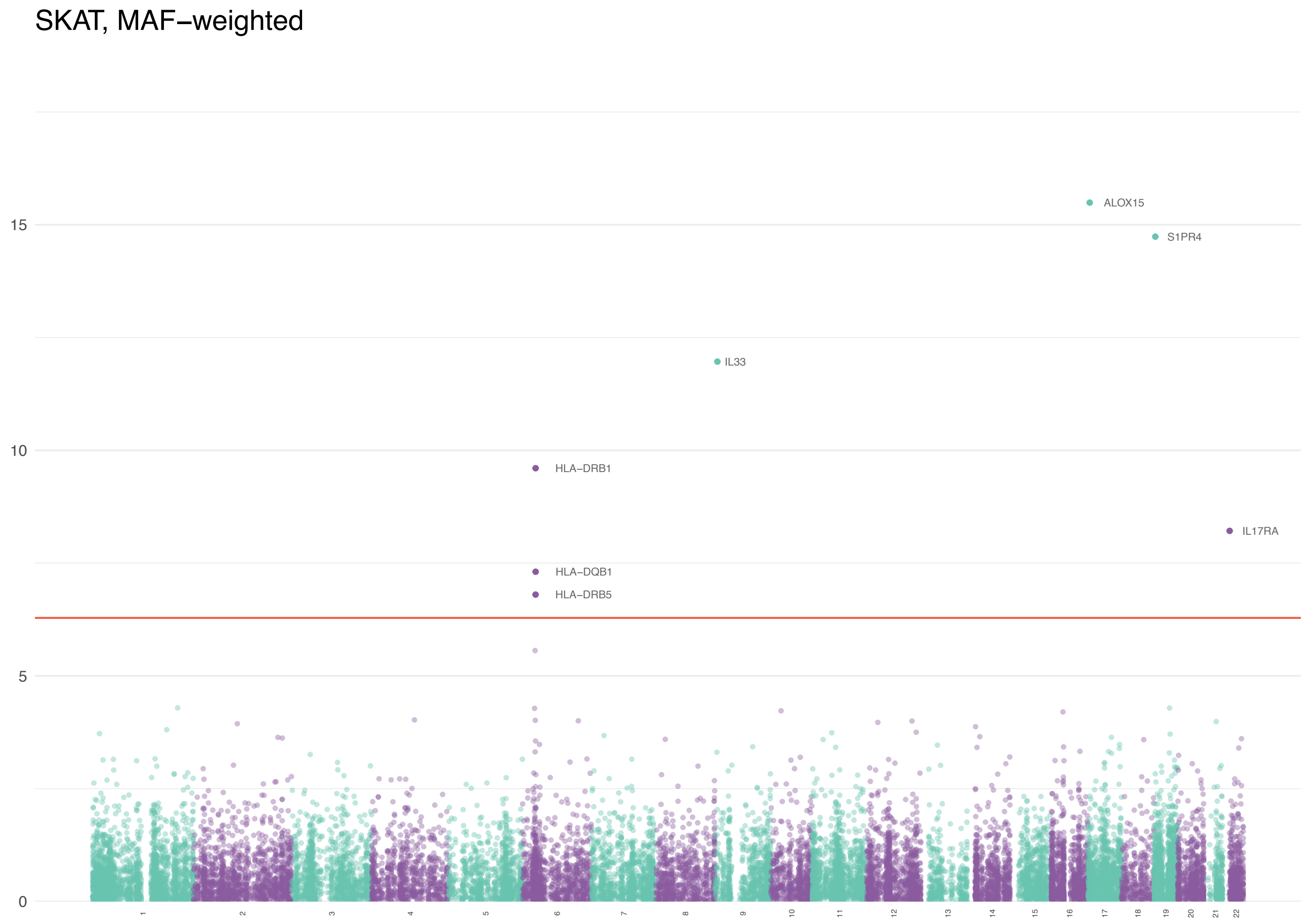

Fig. S5. Results from the MAF-weighted SKAT gene-based analysis. Results from gene-based tests of all canonical transcripts, with chromosomal location at the x-axis and -log10(P) on the y-axis. Genome-wide significance threshold is set at P = 5.18×10^{-7} (-log10(P) = 6.29). The top two most significant genes at each chromosome are labelled with their gene name.

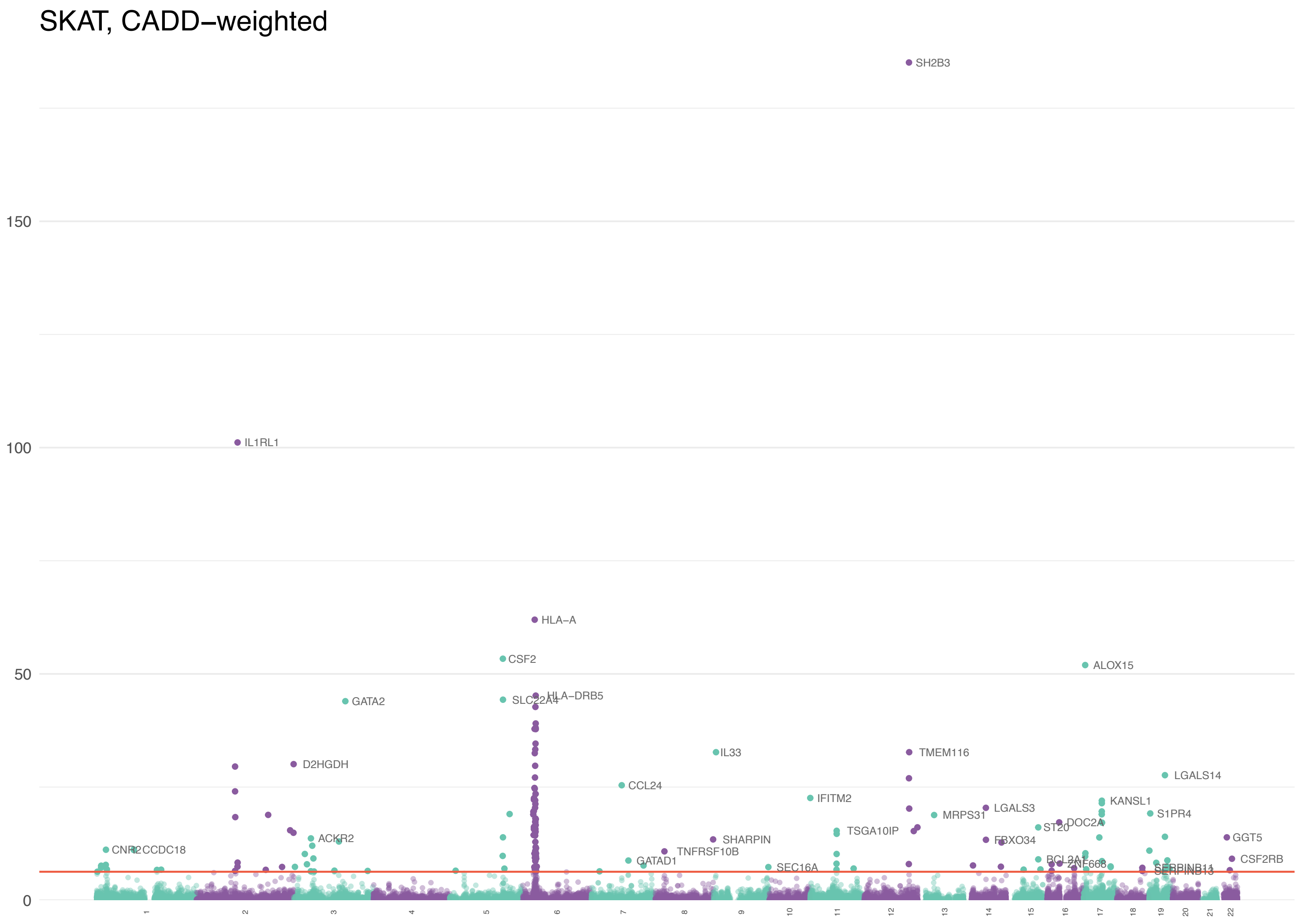

Fig. S6. Results from the CADD-weighted SKAT gene-based analysis. Results from gene-based tests of all canonical transcripts, with chromosomal location at the x-axis and -log10(P) on the y-axis. Genome-wide significance threshold is set at P = 5.18x10^{-7} (-log10(P) = 6.29). The top two most significant genes at each chromosome are labelled with their gene name.

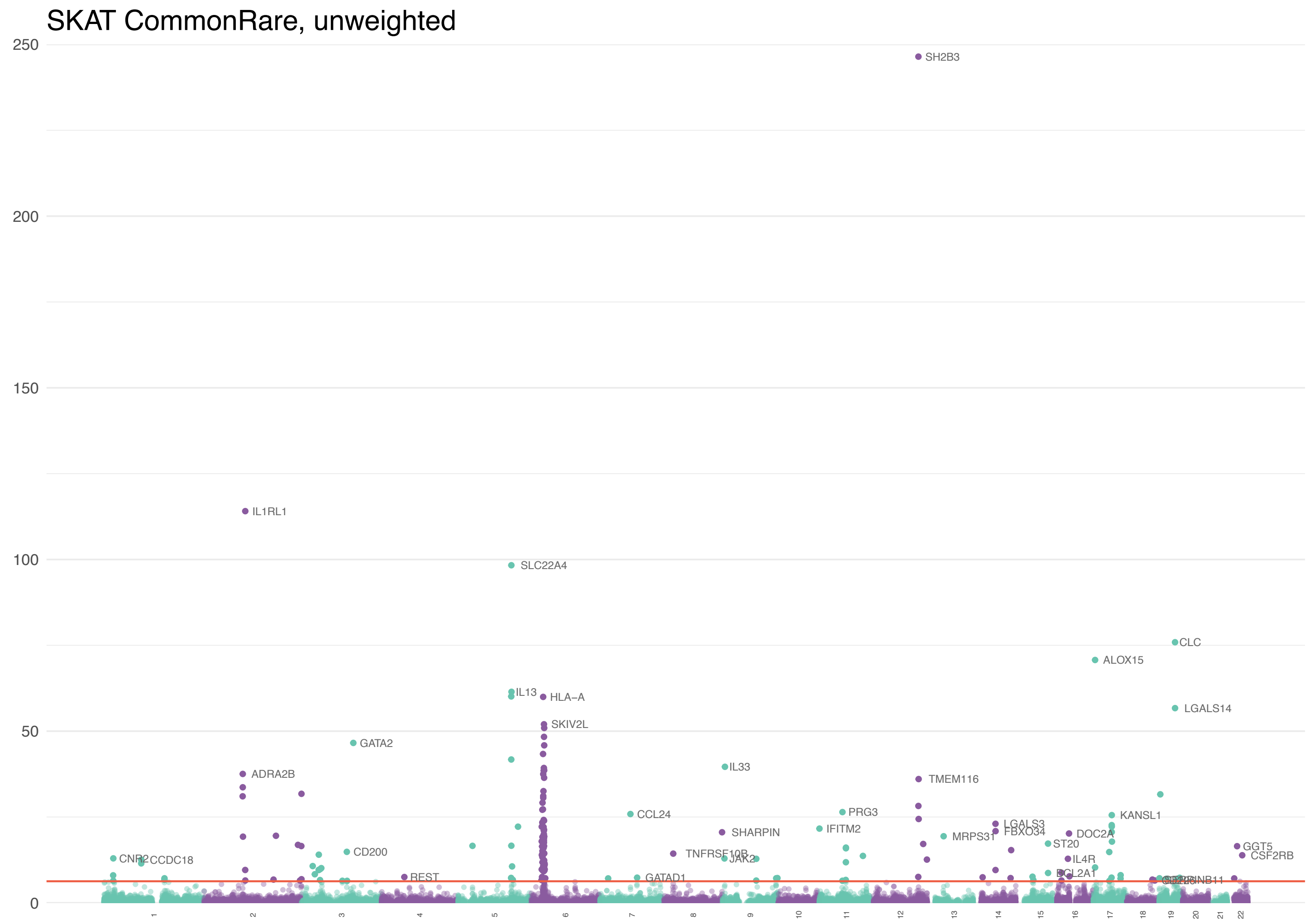

Fig. S7. Results from the unweighted SKAT CommonRare gene-based analysis. The default MAF-threshold of 0.0016 (0.16%) is used as a threshold between common and rare variants. Results from gene-based tests of all canonical transcripts, with chromosomal location at the x-axis and -log10(P) on the y-axis. Genome-wide significance threshold is set at P = 5.18×10^{-7} (-log10(P) = 6.29). The top two most significant genes at each chromosome are labelled with their gene name.

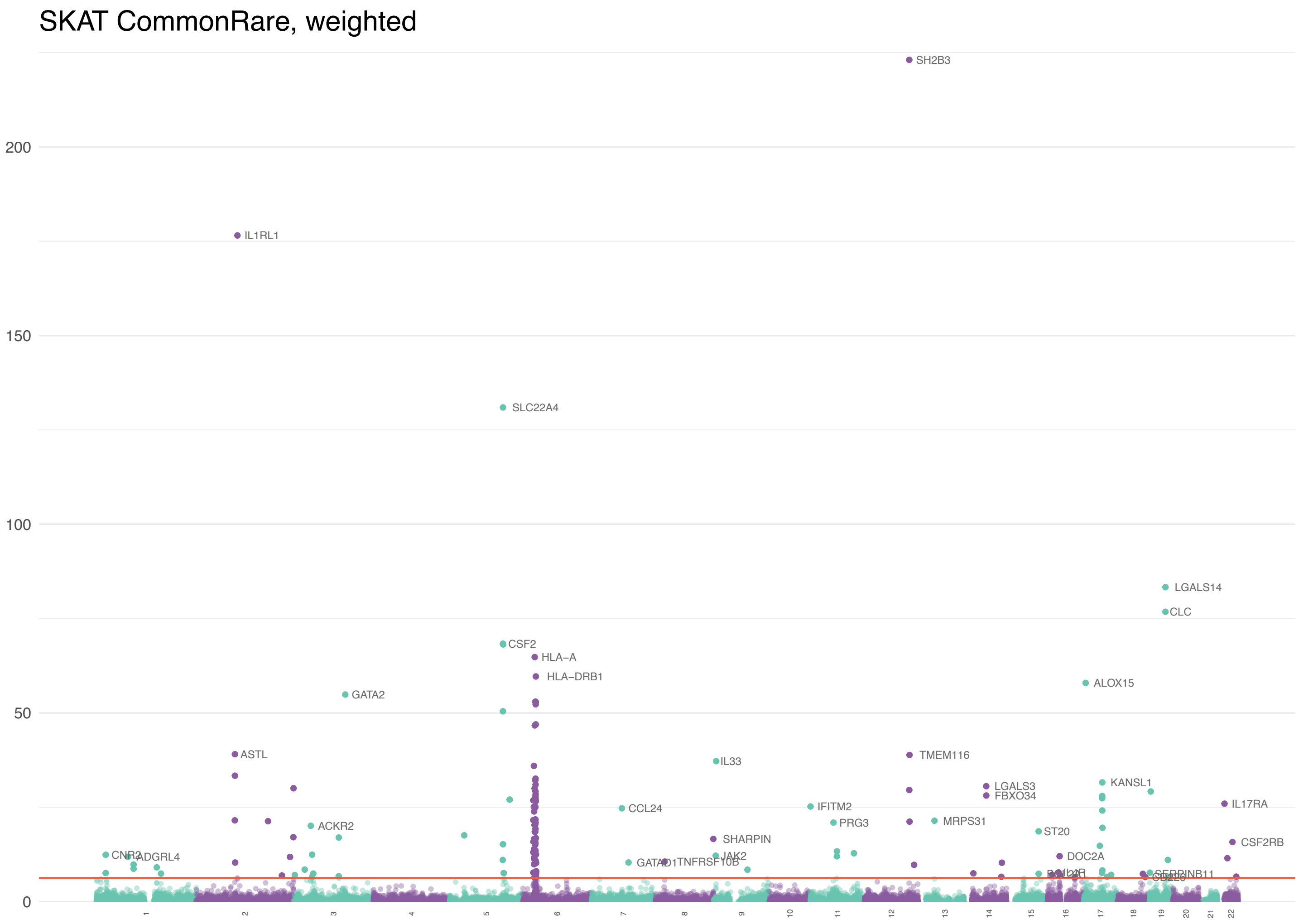

Fig. S8. Results from the weighted SKAT CommonRare gene-based analysis. A MAF-threshold of 0.00025 (0.025%) corresponding to a MAC of 100 is used as a threshold between common and rare variants. Results from gene-based tests of all canonical transcripts, with chromosomal location at the x-axis and -log10(P) on the y-axis. Genome-wide significance threshold is set at P = 5.18x10^{-7} (-log10(P) = 6.29). The top two most significant genes at each chromosome are labelled with their gene name.

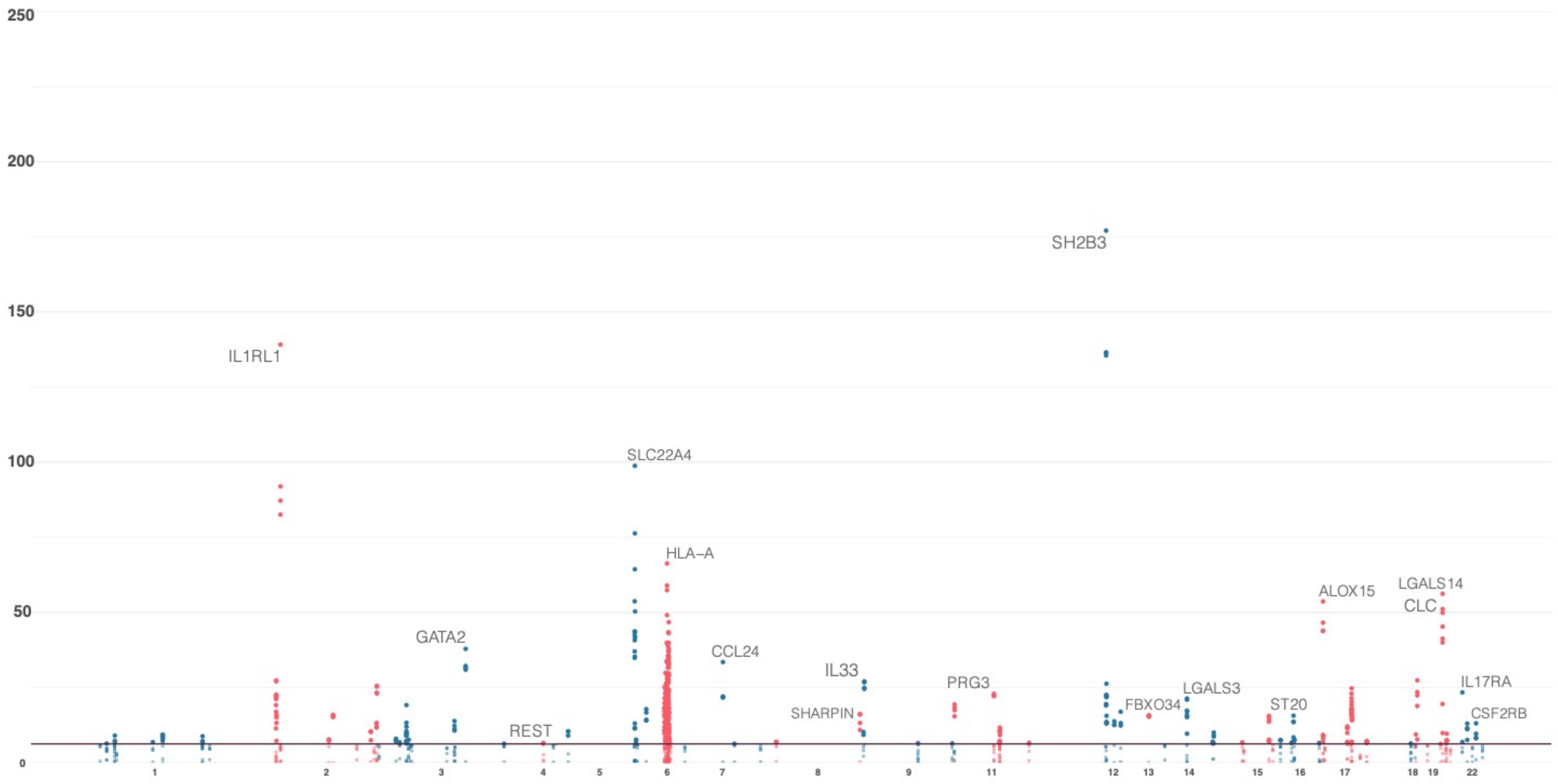

Fig. S9. Significant genes in the SKAT sensitivity analyses; combined results from the fives models of SKAT gene-based analysis. A MAF-threshold of 0.00025 (0.025%) corresponding to a MAC of 100 is used as a threshold between common and rare variants in the weighted CommonRare analysis, and a threshold of $\frac{1}{\sqrt{2 \times sample\ size}}$ = 0.0016 (0.16%) in the unweighted. Results from gene-based tests of all canonical transcripts, with chromosomal location at the x-axis and -log10(P) on the y-axis. Genome-wide significance threshold is set at 5.18×10^{-7} (6.29). These 220 genes correspond to the genes that had a significant association with at least one model. SKAT on significant genes from primary analyses; sensitivity analysis.

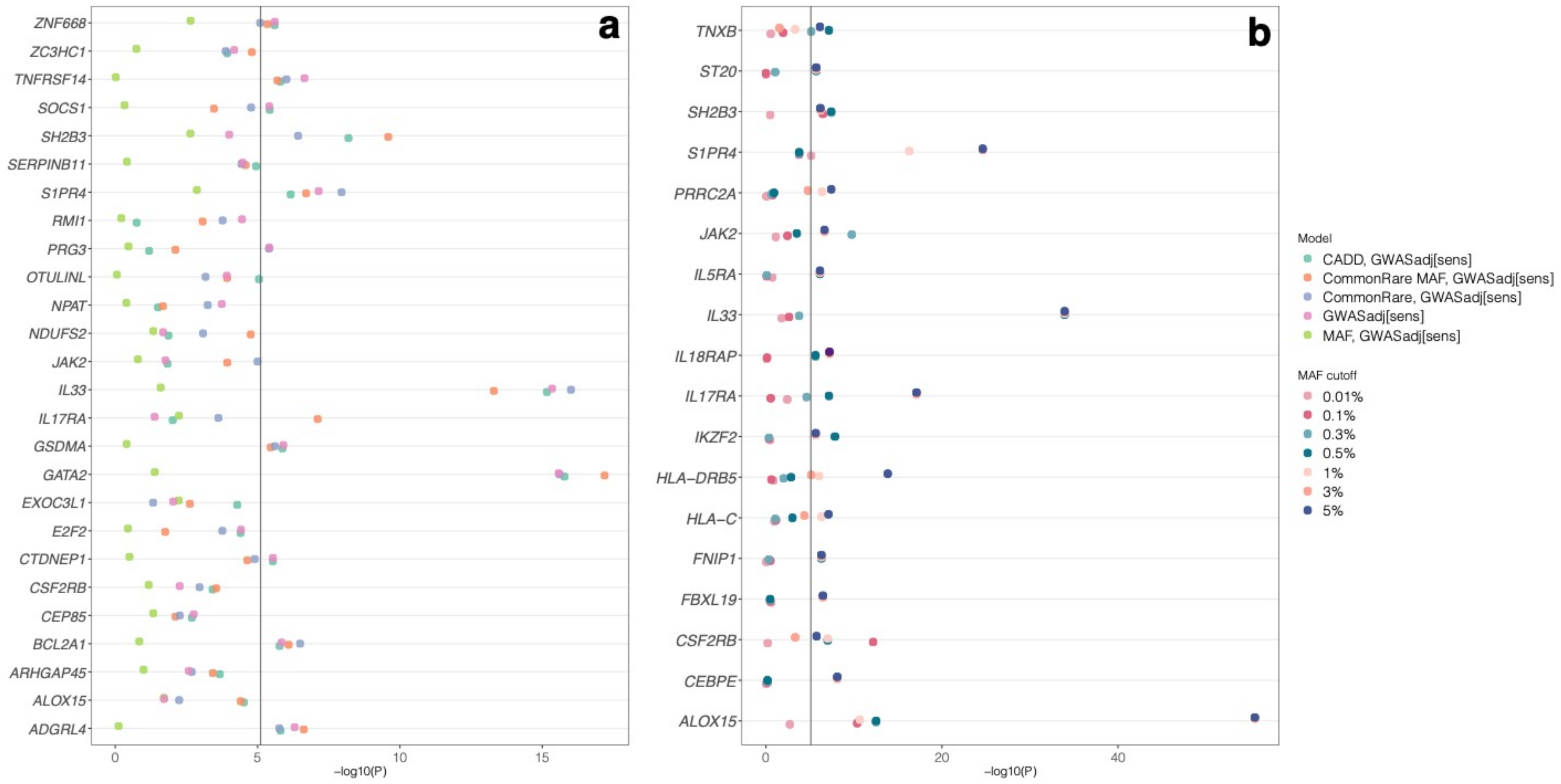

Fig. S10. SKAT P-values from genes that are significant after adjusting for lead-GWAS SNPs, or in the rare only analyses. Gene name is stated on the y axes and -log(P value) on the x axis. The different colors depict the different models used in the analyses. **a)** The five weighting schemes and the 26 genes that were significant after adjusting for lead-GWAS SNPs, with P-values from the sensitivity analyses in the unrelated white-British subset. **b)** Shows the results from when only analyzing rare variants, with different rare variant cut-offs (from 0.01% to 5%). Among all 220 genes that were identified in the primary SKAT analyses, only the 18 that were significant for any of the rare variant cut-offs are shown, others can be found in Supplementary Table S6. In all analyses a P- value cut-off of 5.11×10^{-06} (-log10(P) = 5.11) was used, correcting for the 220 genes times 29 different models tested: seven rare variant cut-offs * two strata (full cohort and unrelated white British) and five weighting schemes for the GWAS-adjusted analyses * two strata, and the five weighting schemes the non-GWAS adjusted (primary/discovery) analyses in the full cohort.

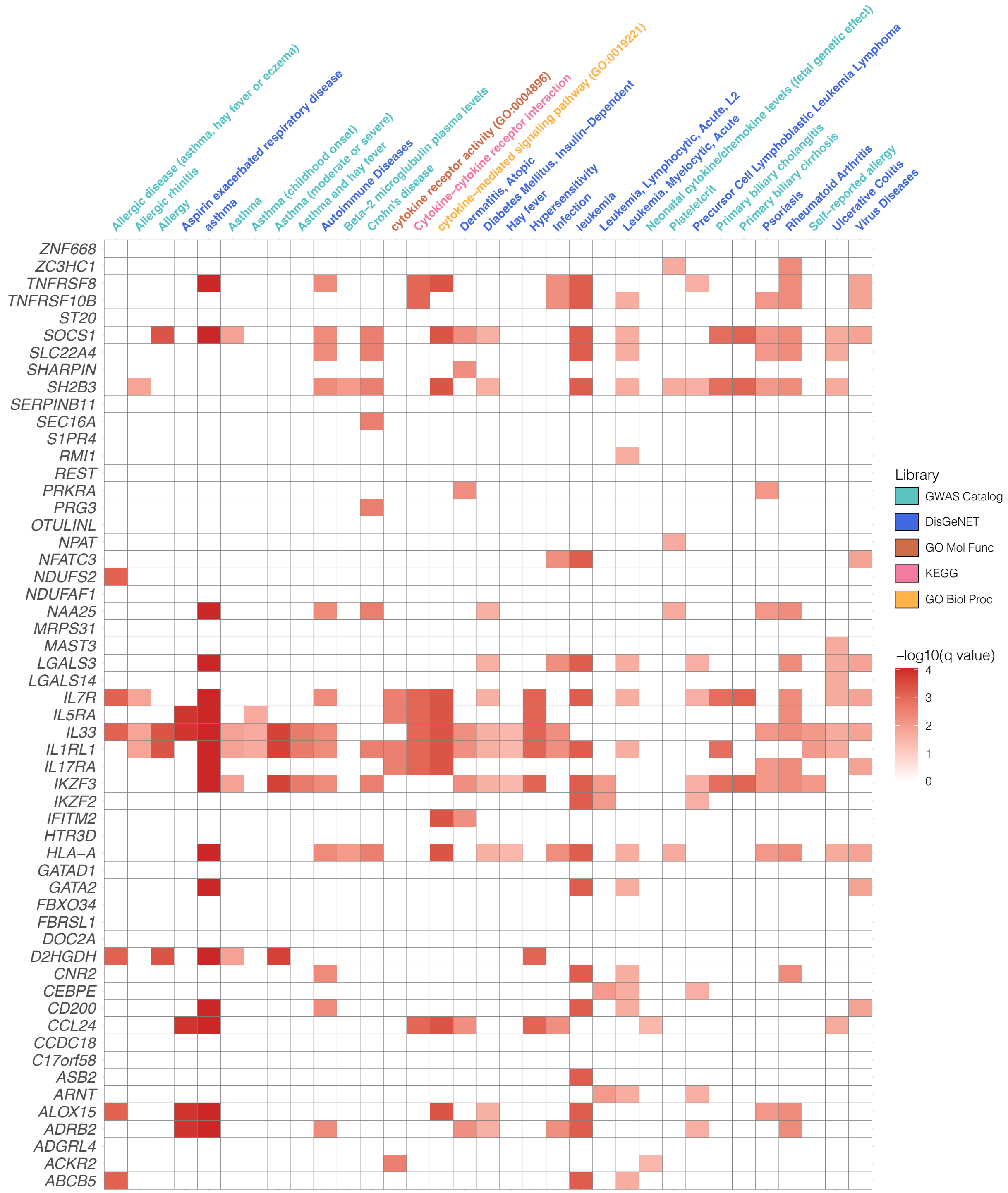

Fig. S11. Overrepresented genes presented as a heatmap. The y-axis shows the genes from our results, which were included in the overrepresented sets, and the x-axis shows the name of the pathways or disease. The queries are colored based on the library in which it is included. Enrichment is visualized in red, with Bonferroni adjusted -log10(q values). Only pathways and traits that are not directly connected to hematopoietic traits such as eosinophil count and eosinophil percentage of white blood cells are represented in the graph.

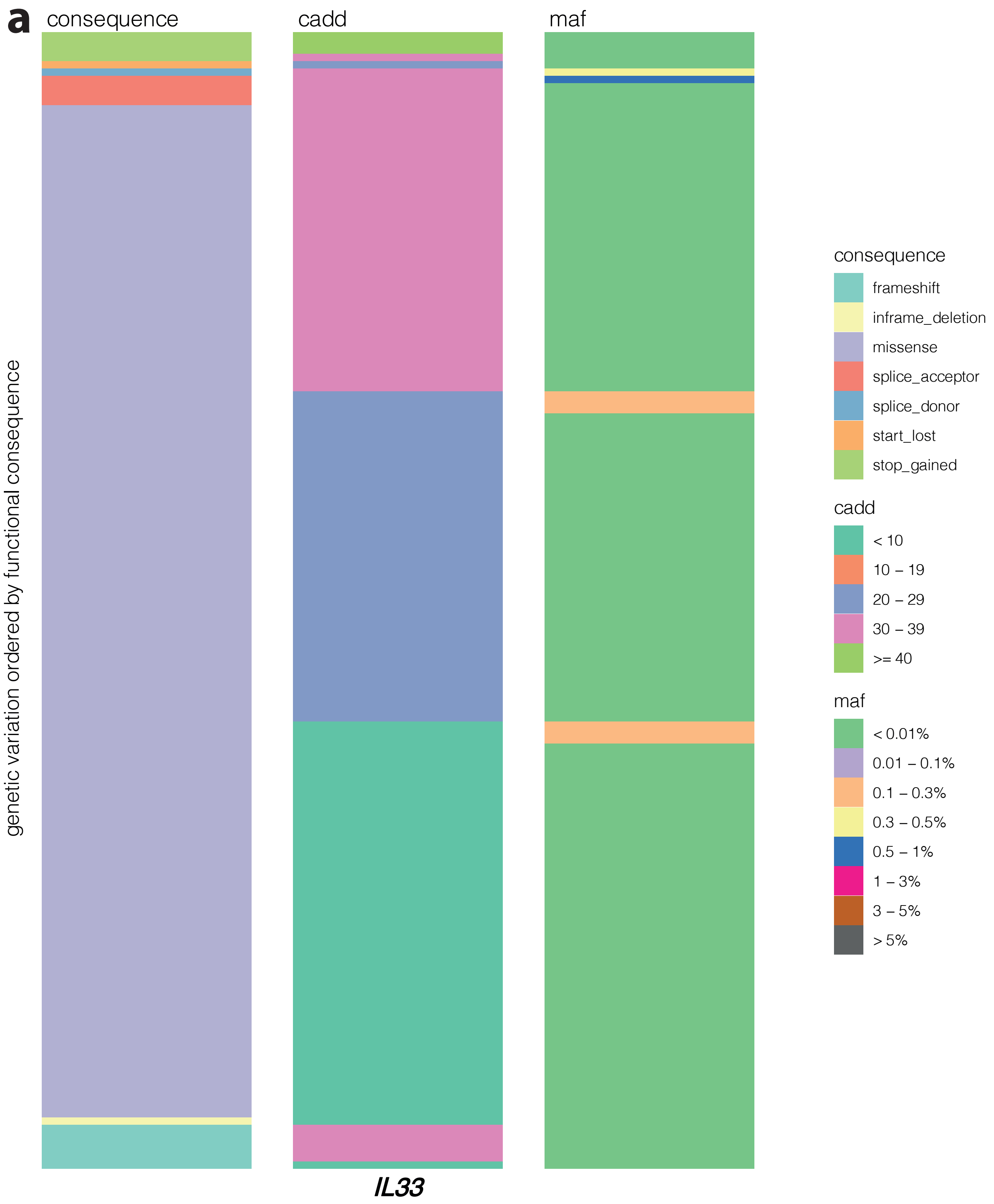
a
consequence
cadd
maf
genetic variation ordered by functional consequence
IL33
consequence
frameshift
inframe_deletion
missense
splice_acceptor
splice_donor
start_lost
stop_gained
cadd
< 10
10 – 19
20 – 29
30 – 39
>= 40
maf
< 0.01%
0.01 – 0.1%
0.1 – 0.3%
0.3 – 0.5%
0.5 – 1%
1 – 3%
3 – 5%
> 5%

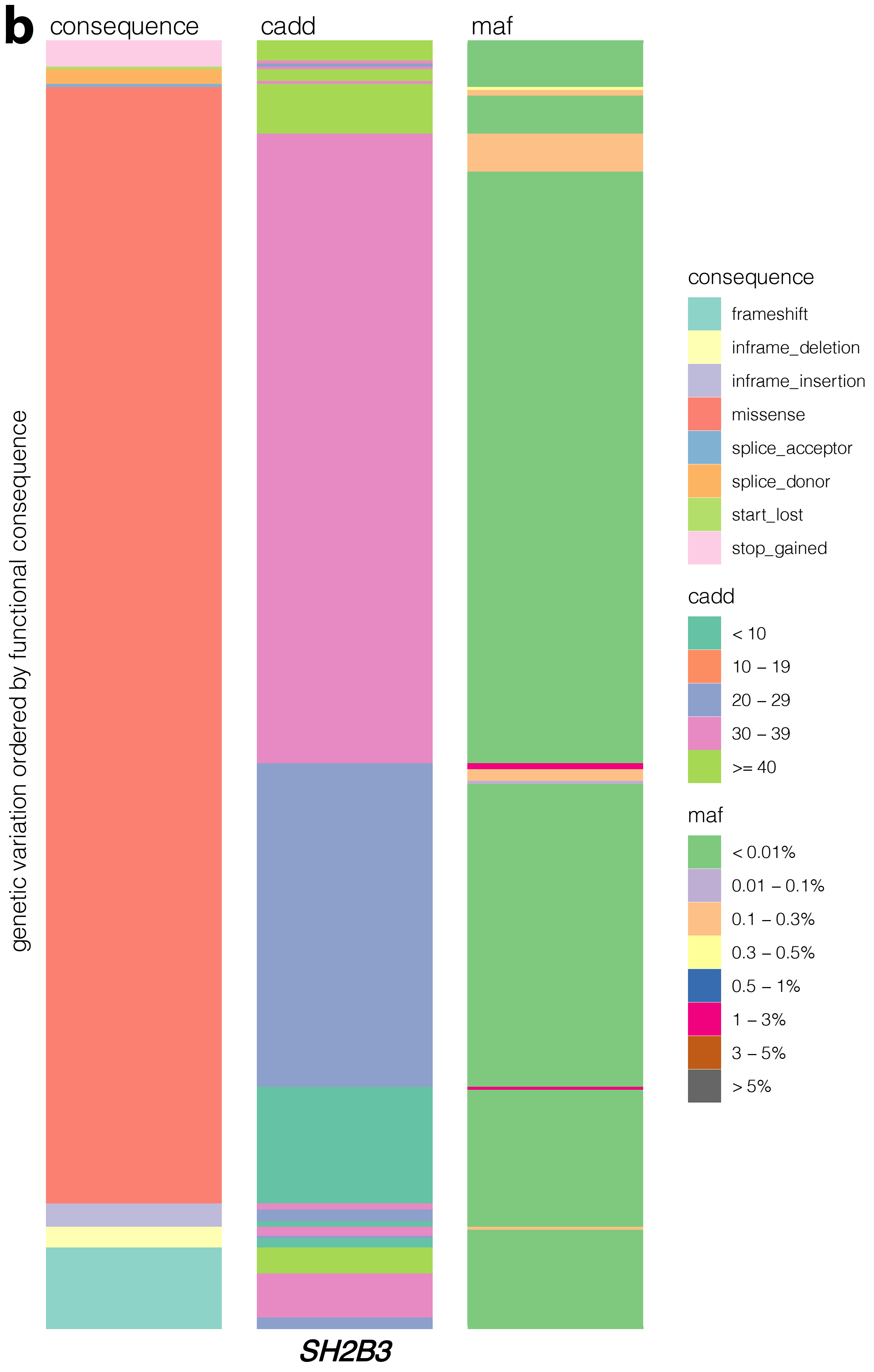
b
consequence
cadd
maf
genetic variation ordered by functional consequence
SH2B3
consequence
frameshift
inframe_deletion
inframe_insertion
missense
splice_acceptor
splice_donor
start_lost
stop_gained
cadd
< 10
10 – 19
20 – 29
30 – 39
>= 40
maf
< 0.01%
0.01 – 0.1%
0.1 – 0.3%
0.3 – 0.5%
0.5 – 1%
1 – 3%
3 – 5%
> 5%

Fig. S12. Illustration of the variant consequence, deleteriousness and frequency distribution in two genes, *IL33* and *SH2B3*. Each bar represents a classification, with variant consequence (left), CADD score bins (middle) and MAF bins (right). The same frequency bins as in the RareOnly analysis have been used. Each vertical line represents a variant, across all three bars, with corresponding consequence, CADD, and frequency values. CADD and frequency bins without any variants represented in these particular genes, are still presented in the legend. **a)** Variant distribution in *IL33*. **b)** Variant distribution in *SH2B3*.

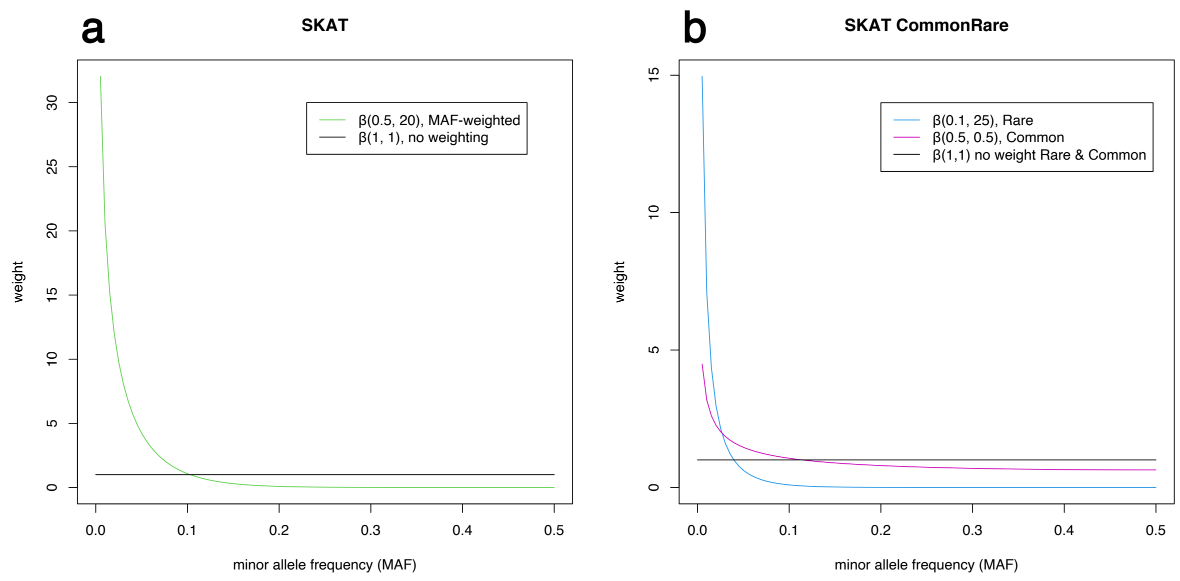

Fig. S13. Weight distributions for the weighting used in the four analyses weighting on MAF. a) Weights used in the SKAT analyses: β(1,1), without any weights and β(0.5, 20), heavily upweighting rare variants. b) Weights used in the SKAT CommonRare analyses: β(1,1), without any weights neither for rare, nor common variants, and β(0.1, 25) for rare variants and β(0.5, 0.5) for common.
